# Cardiovascular and kidney outcomes of GLP‐1 receptor agonists in adults with obesity: A target trial emulation study

**DOI:** 10.1111/dom.70054

**Published:** 2025-08-28

**Authors:** Huilin Tang, Yiwen Lu, Bingyu Zhang, Ting Zhou, Dazheng Zhang, Jiajie Chen, Yong Chen, David A. Asch, Yong Chen

**Affiliations:** ^1^ The Center for Health AI and Synthesis of Evidence (CHASE) University of Pennsylvania Philadelphia Pennsylvania USA; ^2^ Department of Biostatistics, Epidemiology, and Informatics University of Pennsylvania Perelman School of Medicine Philadelphia Pennsylvania USA; ^3^ The Graduate Group in Applied Mathematics and Computational Science, School of Arts and Sciences University of Pennsylvania Philadelphia Pennsylvania USA; ^4^ Pfizer Inc. New York City New York USA; ^5^ Leonard Davis Institute of Health Economics University of Pennsylvania Philadelphia Pennsylvania USA; ^6^ Wharton School University of Pennsylvania Philadelphia Pennsylvania USA; ^7^ Division of General Internal Medicine, Department of Medicine, Perelman School of Medicine University of Pennsylvania Philadelphia Pennsylvania USA; ^8^ Penn Medicine Center for Evidence‐Based Practice (CEP) University of Pennsylvania Philadelphia Pennsylvania USA; ^9^ Penn Institute for Biomedical Informatics (IBI) University of Pennsylvania Philadelphia Pennsylvania USA

**Keywords:** anti‐obesity medication, GLP‐1 receptor agonists, major adverse cardiovascular events, major adverse kidney events

## Abstract

**Aims:**

Glucagon‐like peptide‐1 receptor agonists (GLP‐1RAs) provide cardiovascular and renal benefits in type 2 diabetes, but their real‐world effects in individuals with obesity without diabetes are unclear. This study aimed to evaluate the cardiovascular and kidney outcomes of GLP‐1RAs versus other anti‐obesity medications (AOMs) in adults with obesity.

**Materials and Methods:**

This target trial emulation used TriNetX electronic health records (July 2021–May 2025) to identify adults with obesity and no diabetes history who initiated GLP‐1RAs (liraglutide, semaglutide, or tirzepatide) or other AOMs. Outcomes included major adverse cardiovascular events (MACE), major adverse kidney events (MAKE), all‐cause mortality, mental health conditions, and safety outcomes. Propensity score matching (1:1) balanced baseline characteristics; Cox models estimated hazard ratios (HRs) with 95% confidence intervals (CIs).

**Results:**

The study included 140 169 matched pairs of GLP‐1RA and AOM initiators. Mean follow‐up was 392 days for GLP‐1RA users and 564 days for AOM users. Compared to AOMs, GLP‐1RA use was associated with lower risks of MACE (HR, 0.76; 95% CI, 0.72–0.81), MAKE (HR, 0.64; 95% CI, 0.55–0.74), and all‐cause mortality (HR, 0.49; 95% CI, 0.42–0.57). Mental health outcomes also improved, with reduced risks of depression (HR, 0.63), suicidal ideation/attempt (HR, 0.42), and substance use disorder (HR, 0.58). GLP‐1RAs were not associated with increased risks of acute pancreatitis (HR, 1.17), hypoglycemia (HR, 1.08), or gastrointestinal symptoms (HR, 0.99).

**Conclusions:**

Among adults with obesity but without diabetes, GLP‐1RA use was associated with reduced cardiovascular, kidney, mortality, and mental health risks compared to other AOMs.

## 1 INTRODUCTION

Obesity is a major public health concern, affecting over 890 million adults worldwide and contributing to a wide range of chronic conditions, including type 2 diabetes (T2D), cardiovascular disease (CVD), chronic kidney disease (CKD), and mental health disorders.^1^, ^2^, ^3^, ^4^ Glucagon‐like peptide‐1 receptor agonists (GLP‐1RAs) have emerged as a promising class of medications for obesity management due to their ability to promote weight loss through appetite suppression and delayed gastric emptying.^5^, ^6^, ^7^ Beyond their role in weight control, GLP‐1RAs have demonstrated cardiovascular and kidney benefits in patients with T2D, leading to their widespread use in diabetes management.^8^, ^9^ However, evidence regarding the long‐term effectiveness and safety of GLP‐1RAs in individuals with obesity who do not have preexisting diabetes remains limited. Most studies have focused primarily on short‐term weight loss outcomes, with relatively little attention paid to long‐term cardiovascular, kidney, and mental health outcomes.^5^, ^6^, ^7^

To address this evidence gap, we conducted a target trial emulation using electronic health record (EHR) data from TriNetX to compare the effectiveness and safety of GLP‐1RAs with other anti‐obesity medications (AOMs) in adults with obesity without preexisting diabetes. Specifically, we evaluated the impact of GLP‐1RA use on the incidence of cardiovascular, kidney, and mental health outcomes, as well as their safety profile.

## 2 METHODS

### 2.1 Study design and data source

This study was designed as a target trial emulation, a methodological approach that leverages observational data to approximate the design and analysis of a hypothetical randomized controlled trial (RCT).^10^ By emulating a target trial, we aimed to minimise biases inherent in observational studies while providing robust evidence on the real‐world effectiveness and safety of GLP‐1RAs in adults with obesity. Details of target trial emulation are provided in Table S1.

The study used de‐identified EHR data from the TriNetX database, a large‐scale, federated network of healthcare organizations that provides real‐world clinical data from diverse populations.^11^ The dataset spanned the period from July 2021 (Semaglutide approved for obesity management in June 2021) to May 2025, ensuring a sufficient sample size and follow‐up duration to capture long‐term outcomes. TriNetX includes comprehensive information on demographics, diagnoses, medications, laboratory results, procedures, and outcomes. Informed consent was waived because the study used de‐identified secondary data. This study followed the Strengthening the Reporting of Observational Studies in Epidemiology (STROBE) reporting guidelines.

### 2.2 Study population

We identified adults aged ≥18 years with obesity, defined by either a documented body mass index (BMI) ≥30 kg/m^2^ or the presence of relevant International Classification of Diseases, Tenth Revision, Clinical Modification (ICD‐10‐CM) codes (E66.01, Z68.3, or Z68.4) recorded during a physical examination within the one‐year baseline period prior to treatment initiation (index date). To minimise the potential confounding by indication, we excluded patients with a diagnosis of diabetes, prescription of any glucose‐lowering drugs (insulin, biguanides, sodium‐glucose‐cotransporter 2 inhibitors, dipeptidyl peptidase 4 inhibitors, sulfonylureas, alpha‐glucosidase inhibitors, thiazolidinediones, albiglutide, dulaglutide, exenatide, or lixisenatide), or a haemoglobin A1c (HbA1c) ≥ 6.5%. To ensure a clear temporal relationship between exposure and outcomes, we included only new users of either a GLP‐1RA or an AOM during the study period. The index date was defined as the date of the first prescription of the treatment of interest. We excluded individuals with prior prescriptions of GLP‐1RAs or AOMs, those who initiated both on the index date, those with a history of end‐stage kidney disease (ESKD) or dialysis, and those with a prior diagnosis of the study outcomes. Detailed codes used to define eligibility criteria are provided in Table S2.

### 2.3 Exposure and outcomes

The exposure of interest was the initiation of a GLP‐1RA, including liraglutide, semaglutide, or tirzepatide. The comparator was the initiation of an alternative AOM, including orlistat, naltrexone/bupropion, phentermine/topiramate, or lorcaserin. These agents were selected because they represent real‐world pharmacologic alternatives for obesity treatment, with different mechanisms of action and safety profiles. This comparison allowed for the evaluation of the relative effectiveness and safety of GLP‐1RAs versus other approved medications for obesity management. These drugs were identified using Anatomical Therapeutic Chemical (ATC) or RxNorm codes, as shown in Table S2.

Following an intention‐to‐treat approach, individuals were analysed based on their initial treatment assignment. Follow‐up began 90 days after the index date and continued until the first occurrence of the outcome, death, last clinical encounter, or end of the study period. This strategy helps mitigate the impact of early events and reduces potential bias from reverse causation or early risk selection. The primary outcomes included the incidence of major adverse cardiovascular events (MACE, including acute coronary syndrome, heart failure, and stroke), major adverse kidney events (MAKE, including end‐stage kidney disease, dialysis, and estimated glomerular filtration rate [eGFR] < 15 mL/min/1.73 m^2^), and all‐cause mortality. Secondary outcomes were the incidence of acute coronary syndrome, heart failure, stroke, acute kidney injury, mental health outcomes (e.g., suicidal ideation/attempt, depression, and substance use disorder), and other safety outcomes (including acute pancreatitis, hypoglycemia, and gastrointestinal symptoms). Details on diagnosis codes are presented in Table S2.

### 2.4 Baseline covariates

Baseline demographic covariates were collected during the 1‐year period prior to the index date, including age, sex, race, ethnicity, BMI, baseline comorbidities, prior medication use, healthcare utilisation patterns, and clinical laboratory (Table 1). In the TriNetX network, missing values for sex, race, or ethnicity were categorised as “unknown” and retained in the analysis. Patients with missing data for other covariates required for inclusion/exclusion criteria or propensity score matching (PSM) were automatically excluded, as no imputation was performed.

**TABLE 1:** Baseline patient characteristics before and after propensity score matching.

	Before PSM			After PSM		
Characteristic	GLP‐1RAs (*n* = 174 946)	Other AOMs (*n* = 197 057)	SMD	GLP‐1RAs (140169)	Other AOMs (140169)	SMD
Age at Index, mean (SD), years	48.87 (13.80)	45.74 (14.77)	0.219	47.58 (13.76)	47.69 (14.60)	0.008
Sex						
Female	67.60%	71.27%	0.080	69.94%	69.95%	0.000
Male	27.29%	22.89%	0.102	24.63%	24.59%	0.001
Unknown	5.11%	5.84%	0.032	5.43%	5.46%	0.001
Ethnicity						
Hispanic or Latino	6.16%	6.52%	0.015	6.36%	6.37%	0.001
Not Hispanic or Latino	66.74%	63.82%	0.061	64.95%	64.88%	0.001
Unknown	27.09%	29.67%	0.057	28.69%	28.74%	0.001
Race						
White	67.57%	69.70%	0.046	68.14%	68.22%	0.002
Black or African American	15.69%	13.99%	0.048	15.27%	15.26%	0.000
Asian	1.58%	0.90%	0.061	1.19%	1.13%	0.005
American Indian or Alaska Native	0.35%	0.42%	0.011	0.38%	0.39%	0.000
Native Hawaiian or Other Pacific Islander	0.48%	0.27%	0.033	0.34%	0.34%	0.000
Other Race	3.58%	3.48%	0.006	3.63%	3.59%	0.002
Unknown	10.75%	11.24%	0.016	11.06%	11.08%	0.001
Healthcare utilization						
Office or Other Outpatient Services	56.50%	54.65%	0.037	54.39%	54.52%	0.003
Preventive Medicine Services	24.29%	17.35%	0.172	20.11%	20.14%	0.001
Emergency Department Services	7.29%	10.30%	0.106	8.00%	7.95%	0.002
Hospital Inpatient and Observation Care Services	0.93%	1.83%	0.077	1.06%	1.05%	0.001
Co‐morbidities						
Hypertension	30.17%	20.27%	0.229	24.20%	23.88%	0.007
Lipid disorders	26.44%	16.84%	0.235	20.66%	20.41%	0.006
Ischemic heart diseases	3.36%	1.80%	0.098	2.33%	2.27%	0.004
Pulmonary heart disease	0.90%	0.67%	0.026	0.73%	0.73%	0.000
Atrial fibrillation and flutter	2.23%	1.27%	0.073	1.57%	1.58%	0.001
Other forms of heart disease	6.76%	4.63%	0.092	5.32%	5.29%	0.001
Cerebrovascular diseases	0.74%	0.83%	0.010	0.73%	0.71%	0.002
Chronic kidney disease	1.60%	1.29%	0.025	1.39%	1.39%	0.000
Anxiety	14.42%	19.19%	0.128	15.61%	15.67%	0.002
Sleep disorders	15.96%	11.89%	0.118	13.29%	13.18%	0.003
Neoplasms	12.47%	9.74%	0.087	10.98%	10.91%	0.002
Thyroid diseases	11.88%	9.58%	0.074	10.66%	10.68%	0.001
Vitamin D deficiency	11.52%	8.60%	0.097	9.87%	9.75%	0.004
Osteoarthritis	10.61%	8.48%	0.072	9.39%	9.37%	0.001
Chronic lower respiratory diseases	9.42%	8.80%	0.021	8.84%	8.75%	0.003
Mood disorders	4.88%	9.34%	0.174	5.76%	5.82%	0.003
Migraine	4.32%	8.55%	0.173	5.14%	5.10%	0.002
Liver diseases	3.29%	2.00%	0.080	2.51%	2.44%	0.004
Disorders of gallbladder, biliary tract and pancreas	0.86%	0.81%	0.006	0.82%	0.82%	0.001
COVID‐19	3.79%	3.88%	0.004	3.67%	3.66%	0.001
Post COVID‐19 condition	0.31%	0.34%	0.005	0.32%	0.32%	0.001
Problems related to lifestyle	1.11%	1.44%	0.029	1.21%	1.22%	0.001
Persons with potential health hazards related to socioeconomic and psychosocial circumstances	1.15%	1.15%	0.000	1.08%	1.07%	0.001
Co‐medications						
Diuretics	15.35%	10.64%	0.140	12.60%	12.44%	0.005
Beta blocking agents	10.82%	9.51%	0.043	9.53%	9.56%	0.001
Calcium channel blockers	10.21%	6.88%	0.119	8.10%	8.01%	0.003
Agents acting on renin‐angiotensin system	18.28%	11.32%	0.197	14.09%	13.92%	0.005
Lipid modifying agents	14.95%	9.94%	0.152	11.84%	11.66%	0.006
NSAID	20.02%	22.48%	0.060	20.40%	20.42%	0.001
Antidepressants	17.13%	24.28%	0.177	19.07%	19.14%	0.002
Corticosteroids	27.76%	27.99%	0.005	27.27%	27.34%	0.002
Opioids	11.05%	13.86%	0.085	11.74%	11.71%	0.001
Antithrombotic agents	7.17%	7.73%	0.021	6.93%	6.96%	0.001
Thyroid therapy	8.68%	7.09%	0.059	7.91%	7.92%	0.001
Hormones and related agents	4.35%	5.19%	0.039	4.70%	4.79%	0.004
Antineoplastic agents	4.29%	4.06%	0.011	4.13%	4.13%	0.000
Antipsychotics	3.38%	6.47%	0.143	3.94%	3.96%	0.001
COVID‐19 vaccine	5.04%	8.28%	0.130	5.77%	5.69%	0.004
Vital and lab values						
BMI, kg/m^2^, mean (SD)	38.62 (7.02)	36.86 (6.62)	0.259	38.13 (6.97)	37.67 (6.82)	0.067
≥ 30 and < 35 kg/m^2^	28.73%	37.87%	0.195	31.49%	31.47%	0.001
≥ 35 and < 40 kg/m^2^	26.62%	24.70%	0.044	25.64%	25.78%	0.003
≥ 40 kg/m^2^	28.68%	21.90%	0.157	25.34%	25.53%	0.004
DBP, mmHg, mean (SD)	79.32 (10.66)	78.28 (11.48)	0.093	79.13 (10.68)	78.64 (11.35)	0.045
≥ 90, mmHg	24.48%	23.26%	0.029	23.15%	23.07%	0.002
SBP, mmHg, mean (SD)	128.69 (15.91)	126.63 (16.50)	0.127	128.10 (15.87)	127.49 (16.46)	0.038
≥ 130, mmHg	51.94%	49.14%	0.056	49.40%	49.30%	0.002
eGFR, mL/min/1.73 m^2^, mean (SD)	84.73 (20.35)	84.39 (22.16)	0.016	85.48 (20.61)	83.62 (21.62)	0.088
≥ 45, mL/min/1.73 m^2^	53.56%	43.83%	0.196	47.63%	47.69%	0.001
HDL, mg/dL	47.89 (17.63)	47.41 (18.60)	0.026	47.84 (17.45)	47.54 (18.84)	0.016
≥ 50 mg/dL	22.70%	15.30%	0.190	18.61%	18.52%	0.002
Triglyceride	135.73 (80.50)	135.91 (84.09)	0.002	133.51 (79.84)	136.67 (84.30)	0.039
≥ 150 mg/dL	14.89%	9.86%	0.153	11.89%	11.86%	0.001
LDL, mg/dL	113.17 (33.97)	112.99 (33.39)	0.005	113.26 (33.24)	113.45 (33.73)	0.006
≥ 190 mg/dL	1.07%	0.66%	0.045	0.83%	0.82%	0.001
Cholesterol, mg/dL	189.99 (39.26)	190.17 (39.13)	0.005	189.50 (38.62)	191.08 (39.44)	0.040
≥ 200 mg/dL	17.35%	11.48%	0.168	14.04%	13.97%	0.002
Haemoglobin A1c (HbA1c), %	5.55 (0.40)	5.45 (0.41)	0.259	5.52 (0.41)	5.47 (0.41)	0.132
≥ 6.5%	0.01%	0.01%	0.006	0.01%	0.01%	0.001

Abbreviations: AOMs, anti‐obesity medications; BMI, body mass index; DBP, diastolic blood pressure; eGFR, estimated glomerular filtration rate; GLP‐1RAs, glucagon‐like peptide‐1 receptor agonists; HDL, high‐density lipoprotein; LDL, low‐density lipoprotein; NSAIDs, non‐steroidal anti‐inflammatory drugs; PSM, propensity score matching; SBP, systolic blood pressure; SMD, standard mean difference.

### 2.5 Statistical analysis

We used 1:1 PSM to mitigate potential confounding and ensure comparability between the GLP‐1RA and AOM groups. These propensity scores were estimated using multivariate logistic regression models that included covariates such as age, sex, race/ethnicity, baseline BMI, comorbidities (e.g., hypertension and dyslipidemia), medication use (e.g., antihypertensives and lipid‐modifying agents), and healthcare utilisation patterns (see Table 1). Matching was performed using a nearest‐neighbour algorithm with a calliper width of 0.1 standard deviations of the propensity score. The distribution of baseline covariates between the two groups was assessed using a standard mean difference (SMD), with values less than 0.1 indicating negligible imbalance.^12^

We estimated hazard ratios (HRs) with 95% confidence intervals (CIs) using Cox proportional hazards regression models to compare the risk of outcomes between the matched groups. Kaplan–Meier survival curves were generated to visualize the cumulative incidence of outcomes over time. The proportional hazards assumption was assessed using Schoenfeld residuals models.^13^

### 2.6 Sensitivity and subgroup analyses

Sensitivity analyses were conducted to assess the differential effects of specific types of GLP‐1RAs compared to specific AOMs and to examine associations with follow‐up restricted to 1 year. Subgroup analyses were further performed stratified by age, sex, history of CVD, history of CKD, and baseline BMI. To evaluate potential unmeasured confounding, we assessed the risk of four negative control outcomes (NCOs)—dog bites, nail disorders, hearing test with normal findings, and sebaceous cyst, which are unlikely to be influenced by medication choice, but may be affected by differences in healthcare utilisation and other unmeasured confounders.^14^ Statistical analysis was conducted within the TriNetX platform using the R survival package. A two‐sided *p*‐value less than 0.05 is considered statistically significant.

## 3 RESULTS

### 3.1 Study cohort

**FIGURE 1:**
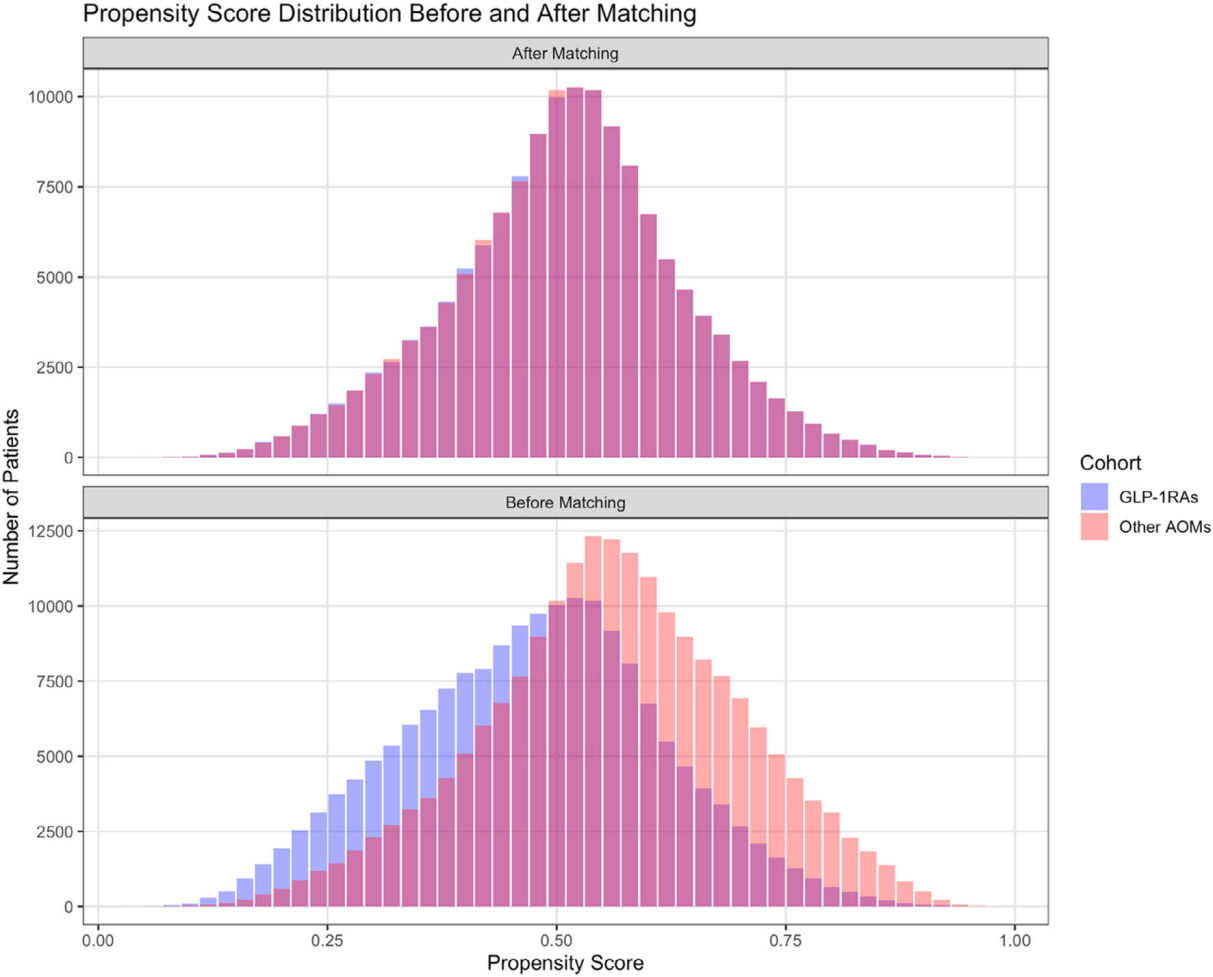
Propensity score distribution for glucagon‐like peptide‐1 receptor agonist (GLP‐1RA) and other anti‐obesity medication before and after propensity score matching.

The study included 174 946 obese adults who initiated GLP‐1RAs and 197 057 adults who initiated other AOMs. Details of cohort construction are presented in Table S3. After applying 1:1 PSM to balance baseline characteristics, each group comprised 140 169 individuals. The matched cohorts were well balanced across demographics, comorbidities, medication use, healthcare utilisation, vital signs, and lab results, with SMD less than 0.1 for all baseline covariates, except for Haemoglobin A1c (Table 1). Among the included individuals, the mean age was 48 years, 71% were women, and 70% were White. The mean follow‐up duration was 392 (standard deviation [SD], 329) days for GLP‐1RA and 564 (SD, 410) days for the other AOM group. The love plots of propensity score distributions before and after matching in the main, sensitivity, and subgroup analyses are present in Figure 1 and Figure S1. The proportional hazards assumption held for most outcomes (Table S4).

### 3.2 Primary outcomes

**FIGURE 2:**
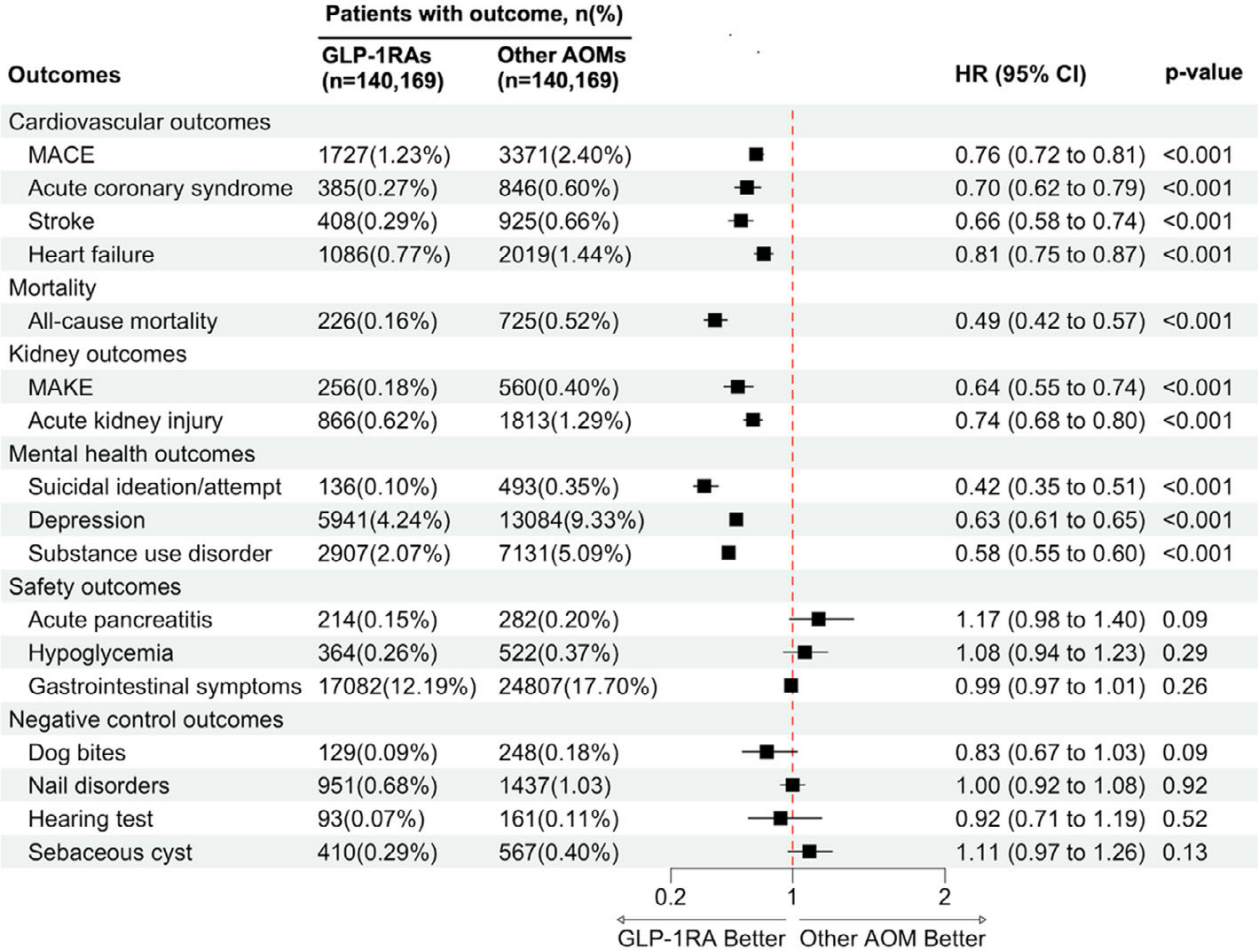
Effectiveness and safety outcomes with glucagon‐like peptide‐1 receptor agonists (GLP‐1RAs) versus other anti‐obesity medications (AOMs) in individuals with obesity. Major adverse cardiovascular events (MACE) including acute coronary syndrome, heart failure, and stroke; major adverse kidney events (MAKE) including end‐stage kidney disease and dialysis; CI, confidence interval; HR, hazard ratio.

**FIGURE 3:**
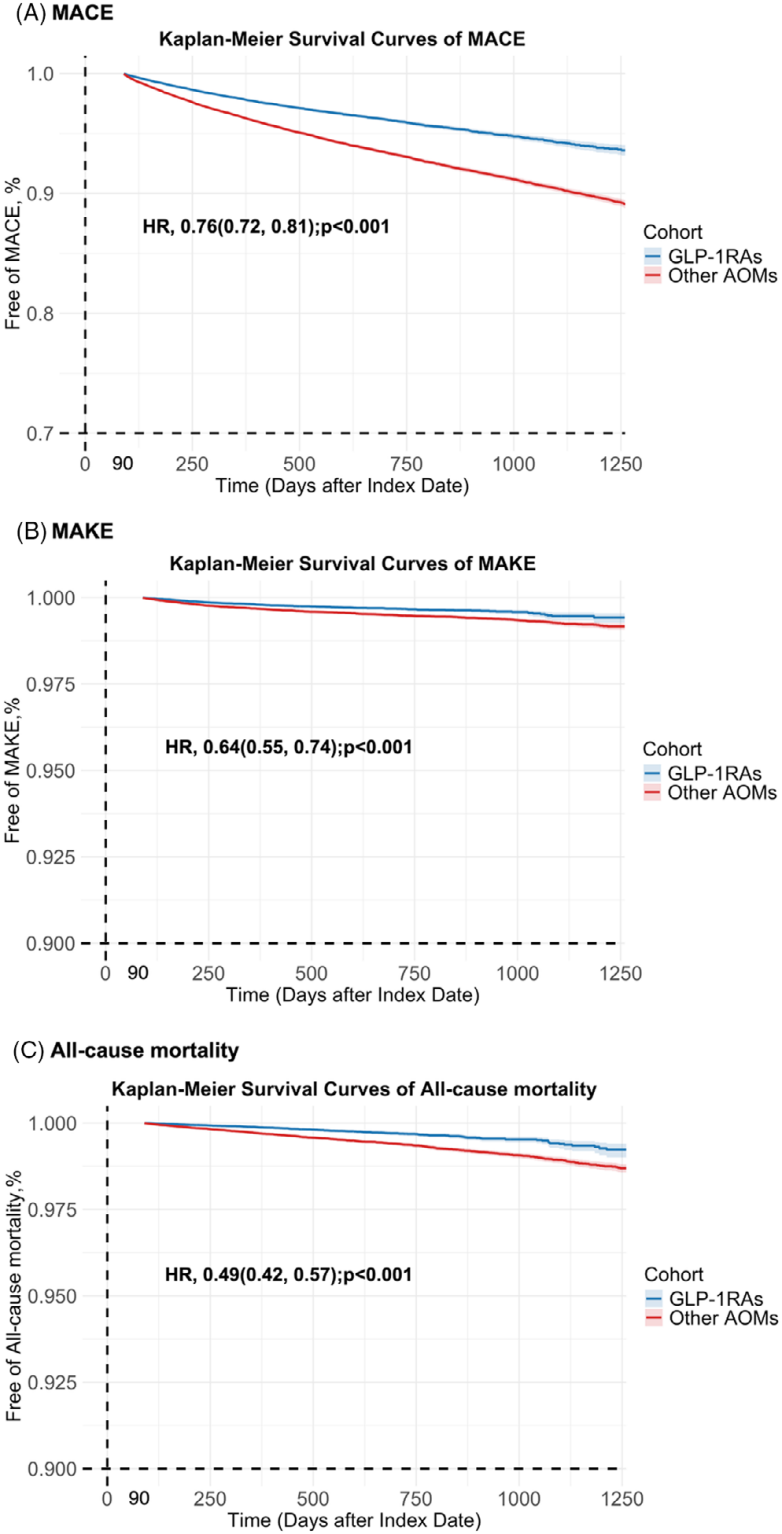
Kaplan–Meier estimates of event‐free rate between GLP‐1RAs and other anti‐obesity medication (AOMs) groups. Major cardiovascular events (MACE), including acute coronary syndrome, heart failure, and stroke; major adverse kidney events (MAKE), including end‐stage kidney disease and dialysis; CI, confidence interval; HR, hazard ratio.

GLP‐1RA use was associated with a significant reduction in the risk of incident MACE (HR, 0.76; 95% CI, 0.72–0.81; *p* < 0.001), MAKE (HR, 0.64; 95% CI, 0.55–0.74; p < 0.001), and all‐cause mortality (HR, 0.49; 95% CI, 0.42–0.57; *p* < 0.001) compared to other AOMs (Figure 2). Kaplan–Meier survival analysis of the above outcomes showed a clear separation in event‐free survival curves between the GLP‐1RA and AOM groups (Figure 3).

### 3.3 Secondary outcomes

GLP‐1RA use was associated with a lower risk of multiple cardiovascular outcomes, including acute coronary syndrome (HR, 0.70; 95% CI, 0.62–0.79; *p* < 0.001), heart failure (HR, 0.81; 95% CI, 0.75–0.87; *p* < 0.001), and stroke (HR, 0.66; 95% CI, 0.58–0.74; *p* < 0.001) (Figure 2). Additionally, GLP‐1RA users had a lower risk of acute kidney injury (HR, 0.74; 95% CI, 0.68–0.80; *p* < 0.001).

GLP‐1RA use was associated with a lower risk of multiple mental health outcomes, including depression (HR, 0.63; 95% CI, 0.61–0.65; p < 0.001) and suicidal ideation/attempt (HR, 0.42; 95% CI, 0.35–0.51; *p* < 0.001) and substance use disorder (HR, 0.58; 95% CI, 0.55–0.60; *p* < 0.001) (Figure 2).

No significant differences were found between GLP‐1RAs and other AOMs in the risk of acute pancreatitis (HR, 1.17; 95% CI, 0.98–1.40; *p* = 0.09), hypoglycemia (HR, 1.08; 95% CI, 0.94–1.23; *p* = 0.29), or gastrointestinal symptoms (HR, 0.99; 95% CI, 0.97–1.01; *p* = 0.26).

### 3.4 Sensitivity and subgroup analyses

As expected, no significant differences were observed for NCOs, including dog bites (HR: 0.83, 95% CI: 0.67–1.03), nail disorders (HR, 1.00; 95% CI, 0.92–1.08), hearing test (HR, 0.92; 95% CI, 0.71–1.19), and sebaceous cyst (HR: 1.11, 95% CI: 0.97, 1.26), suggesting that the association observed in this study is unlikely to be driven by unmeasured confounding or bias (Figure 2). The results remained consistent in sensitivity analyses restricted to a one‐year follow‐up and across comparisons of different GLP‐1RAs versus specific AOMs (Table S5). Subgroup analyses stratified by age, sex, history of CVD or CKD, and baseline BMI also yielded similar results, further reinforcing the robustness of the observed associations (Table S6).

## 4 DISCUSSION

In this large, propensity score‐matched target trial emulation study of obese adults, initiation of GLP‐1RAs was associated with a significantly lower risk of MACE, MAKE, and all‐cause mortality compared to other AOMs. Additionally, GLP‐1RA use was linked to improvements in multiple cardiovascular, kidney, and mental health outcomes, with no significant increase in major safety concerns (e.g., pancreatitis and gastrointestinal symptoms). The results were consistent in the subgroup and sensitivity analyses. These findings add to the growing body of evidence supporting the cardiometabolic benefits of GLP‐1RAs beyond their primary role in weight management.

While cardioprotective and renoprotective effects of GLP‐1RAs have been well established in patients with T2D,^8^, ^9^ our results demonstrate these benefits of GLP‐1RAs in patients with obesity, even in the absence of diabetes, further supporting their role in obesity management. These observed benefits may be attributable to multiple mechanisms, including weight reduction,^5^, ^6^, ^7^ improved insulin sensitivity,^15^, ^16^ reduced systemic inflammation,^17^, ^18^ and enhanced endothelial function.^19^, ^20^ Furthermore, GLP‐1RAs have been shown to lower glomerular hyperfiltration and albuminuria,^21^, ^22^ which may contribute to their renoprotective effects. Given the increasing prevalence of cardiovascular and kidney diseases among individuals with obesity and T2D,^23^ these findings have significant implications for treatment strategies aimed at reducing cardiovascular and kidney morbidity and mortality.

Beyond their well‐established cardiovascular and kidney benefits, this study highlights the potential mental health benefits associated with GLP‐1RA use.^24^ Specifically, we observed significantly lower risk of depression, suicidal ideation/attempt, and substance use disorders among GLP‐1RA users, suggesting possible neuropsychiatric benefits of GLP‐1RAs.^24^, ^25^ These findings are consistent with prior observational studies using the TriNetX platform, which reported similar reductions in risks of suicide and substance use disorders.^26^, ^27^, ^28^ While the underlying mechanisms remain speculative, emerging evidence points to the role of *central GLP‐1 receptor signalling* in modulating mood, reward processing, and addiction pathways.^29^, ^30^ GLP‐1 receptors are expressed in key brain regions such as the *ventral tegmental area* and *nucleus accumbens*,^31^ where they may influence *dopaminergic activity* and behavioural responses related to motivation and mood. These neurobiological actions may underlie the observed mental health effects of GLP‐1RAs. To further explore the biological plausibility of these findings, we compared specific GLP‐1RAs with other centrally acting AOMs. Naltrexone/bupropion exerts its effects through dopamine and norepinephrine reuptake inhibition and μ‐opioid receptor antagonism,^32^ while phentermine/topiramate modulates sympathetic tone and GABAergic activity,^33^ mechanisms also implicated in mood regulation and reward circuitry. These mechanisms strengthen the biological plausibility of GLP‐1RAs. Given the increasing recognition of the bidirectional relationship between obesity and mental health disorders,^34^ GLP‐1RAs may offer a novel therapeutic approach that addresses both metabolic and psychological aspects of obesity. Despite this encouraging speculation, further research is needed to elucidate the neurobiological mechanisms underlying the mental health benefits of GLP‐1RAs and to determine their clinical relevance in populations with preexisting psychiatric disorders.

In this study, GLP‐1RA use was not associated with an increased risk of acute pancreatitis compared to other AOMs, consistent with findings from previous observational studies.^35^, ^36^ Although early safety concerns were raised regarding pancreatitis, more rigorous analyses have largely refuted a causal relationship.^36^ Similarly, while gastrointestinal side effects like nausea and vomiting are common during the initiation of GLP‐1RA therapy,^37^ these symptoms tend to diminish over time and were not significantly different in frequency compared to other AOMs in our study. No significant increase in the risk of hypoglycemia was observed among GLP‐1RA users overall; however, a significant increase was noted in women. Prior study reported an increased risk of hypoglycemia among patients with obesity but without diabetes when comparing GLP‐1RA with placebo.^38^ Although no significant increase in risk of hypoglycemia was observed in patients with obesity, careful monitoring for hypoglycemia remains warranted, especially in patients at elevated risk of hypoglycemia.

This study has several limitations that should be considered when interpreting the findings. First, as with all observational studies, residual confounding cannot be fully ruled out due to the non‐randomized nature of treatment assignment. While we used PSM to balance observed covariates, unmeasured confounders (e.g., socioeconomic status) may still bias the results. We included NCOs, which showed no significant associations, providing indirect support for robustness. However, limited information on time‐varying covariates prevented us from adjusting for time‐varying confounding, which may introduce bias in the estimated effects. Second, our analysis relied on structured data from EHRs, which are subject to inherent limitations such as variability in documentation practices, missing or incomplete data, and potential misclassification of exposures and outcomes.^39^, ^40^ These issues are especially relevant for outcomes such as mental health conditions, gastrointestinal symptoms, and mortality, which may be underreported or inconsistently coded, potentially introducing information bias. Although outcome definitions were based on diagnosis codes that may vary across institutions, potential underreporting of subjective outcomes (such as gastrointestinal symptoms and mental disorders) could have led to differential misclassification. While stratification by healthcare utilization intensity was not feasible, our PS model included multiple utilization measures, including outpatient visits, preventive care, emergency visits, and hospitalisations, which were well‐balanced after matching, suggesting minimal impact of differential misclassification on our findings. Third, we lacked prescribing data on dosage, medication adherence, and treatment duration, limiting our ability to differentiate between prescribing patterns based on indication or dosage, assess the *dose‐dependent effects, and* explore strategies to optimise long‐term use. Fourth, due to analytic constraints within the TriNetX platform, we were unable to calculate incidence rates based on person‐time. As a result, outcomes are reported as cumulative event counts rather than incidence rates, limiting direct comparability with studies that report standardised rates over time. Fifth, the federated nature of the TriNetX database may introduce heterogeneity in diagnostic and prescribing practices across sites. However, site‐level data are not available, and we could not adjust for between‐site variability. This may affect generalisability and introduce unmeasured variability in outcome and exposure classification. Sixth, the mean follow‐up duration differed between groups, with notably shorter follow‐up in the GLP‐1RA group compared to the other AOM group. This imbalance could introduce bias in estimating outcome incidence. However, we used a Cox proportional hazards model, which could account for differences in follow‐up durations between groups. Seventh, the proportional hazards assumption was violated for several outcomes (e.g., all‐cause mortality), suggesting that HRs may vary over time. As a result, these estimates should be interpreted as average effects rather than constant associations throughout the follow‐up period. Finally, although baseline HbA1c showed a slight imbalance after matching (SMD = 0.132), the overall values seemed to be similar (5.52% vs. 5.47%), suggesting that outcome differences are unlikely to be driven solely by HbA1c. Residual differences may reflect the inclusion of patients with diabetes or prediabetes in the GLP‐1RA group, potentially affecting certain outcomes (e.g., cardiovascular and mental outcomes).

In conclusion, among adults with obesity, GLP‐1RA use was associated with lower risks of cardiovascular and kidney events, mortality, and mental health‐related risks compared to other AOMs. These findings add to the growing body of evidence supporting the broader therapeutic benefits of GLP‐1RAs beyond glycemic control and underscore the need for continued evaluation of their long‐term safety and effectiveness across diverse patient populations.

## AUTHOR CONTRIBUTIONS

H.T. and Y.C.(CHASE) conceived the study. H.T. performed the data analysis. H.T. and Y.L. visualised the analysis results. All authors interpreted the results. H.T. drafted the main manuscript. All authors provided critical edits to the early draft and approved the final version of the manuscript. The corresponding author attests that all listed authors meet authorship criteria and that no others meeting the criteria have been omitted.

## FUNDING INFORMATION

This work was supported in part by National Institutes of Health (U01TR003709, U24MH136069, RF1AG077820, R01AG073435, R56AG074604, R01LM013519, R01DK128237, R21AI167418, R21EY034179). The funding institutions had no role in the design and conduct of the study; collection, management, analysis, and interpretation of the data; preparation, review, or approval of the manuscript; and decision to submit the manuscript for publication.

## CONFLICT OF INTEREST STATEMENT

Dr. David A. Asch is a partner and part‐owner of VAL Health and serves on the advisory boards of Thrive Global and Morpheus. Dr. Yong Chen (Pfizer, New York City, NY, USA) reported receiving personal fees from Merck & Co., Inc. and Pfizer Inc. outside the submitted work. All other authors declare no conflict of interest.

## PEER REVIEW

The peer review history for this article is available at https://www.webofscience.com/api/gateway/wos/peer‐review/10.1111/dom.70054.

## Supporting information


**Table S1.** Target trials emulation.
**Table S2.** Codes used to identify outcomes, diseases, and drugs.
**Table S3.** Details of cohort construction.
**Table S4.** Schoenfeld residual test for proportional hazard assumption.
**Table S5.** Sensitivity analyses of specific GLP‐1RAs versus AOMs and associations with 1‐year follow‐up.
**Table S6.** Hazard Ratios (95% CI) from subgroup analyses of GLP‐1RAs versus other AOMs.
**Figure S1.** Love plots of propensity score distributions before and after matching in sensitivity and subgroup analyses.

## Data Availability

The data could be obtained on a reasonable request through TriNetX. Further information about the data of TriNetX can be accessed on their website: https://trinetx.com.
