# Supplementary material for: Cardiovascular and kidney outcomes of GLP‐1 receptor agonists in adults with obesity: A target trial emulation study
Source: Diabetes Obes Metab. 2025 Aug 28;27(11):6527–36. doi: 10.1111/dom.70054 (PMC12515787; doi:10.1111/dom.70054)

**Supplemental materials**

**Table S1**. Target trials emulation

**Table S2.** Codes used to identify outcomes, diseases, and drugs

**Table S3.** Details of cohort construction

**Table S4**. Schoenfeld residual test for proportional hazard assumption.

**Table S5.** Sensitivity analyses of specific GLP-1RAs vs. AOMs and associations with one-year follow-up

**Table S6.** Hazard Ratios (95% CI) from subgroup analyses of GLP-1RAs vs. other AOMs

**Figure S1.** Love plots of propensity score distributions before and after matching in sensitivity and subgroup analyses.

**Table S1**. Target trial emulation

| Approaches | Target trial | Target Trial Emulation |
| --- | --- | --- |
| Eligibility criteria | - Adults (Age ≥ 18 years) with Obesity (defined by body mass index ≥30 kg/m^2^ or diagnosis codes) - No prescription for GLP-1RA or other anti-obesity medications (AOM) within past year - No prior diagnosis of diabetes, any glucose-lowering drug, or HbA1c≥ 6.5% - No contraindication to study drugs (end-stage renal disease/dialysis) - No prior diagnosis of outcomes of interest | Same as for the target trial |
| Treatment strategies | Initiation treatment with a GLP-1RA, or another AOM | The date of initiation treatment was the date of the first prescription of a GLP-1RA or another AOM |
| Treatment assignment | Individuals are randomly assigned to GLP-1RA or another AOM at baseline. Individuals and their treating physicians will be aware of the assigned treatment strategy. | Treatment is not assigned randomly.  Randomization was emulated using 1:1 propensity score matching to address potential confounders. |
| Outcomes | The primary outcomes included incidence of major adverse cardiovascular events, all-cause mortality, and major adverse kidney events.  Secondary outcomes were: 1) incidence of cardiovascular related outcomes including heart failure, acute coronary syndrome, stroke; 2) incidence of kidney outcomes including acute kidney injury; 3) incidence of mental health outcomes including depression, suicidal ideation/attempt and substance use disorder; and 4) incidence of other safety outcomes including acute pancreatitis, hypoglycemia and gastrointestinal symptoms. | Same as for the target trial |
| Follow-up | For each eligible individual, follow-up starts at 90 days after treatment assignment and ends on outcome, death, last clinical encounter, or the end of the study, whichever comes first. | Same as for the target trial |
| Causal contrasts | Intention-to-treat effect | Observational analogs of the intention-to-treat |
| Statistical analysis | Kaplan–Meier survival analysis is used to estimate the survival curves and Cox proportional hazard regression models are used to estimate hazard ratios with 95% CIs by comparing participants assigned to GLP-1RAs versus other AOMs.  Sensitivity analyses were performed by limiting follow-up to 1 year and differential effects of specific types of GLP-1RAs compared to specific AOMs | Same as for the target trial  Negative control outcome analysis is performed |

GLP-1RAs, glucagon-like peptide-1 receptor agonists; AOMs, anti-obesity medications; CI, confidence interval.

**Table S2. codes used to identify outcomes, diseases, and drugs**

| **Category** | **Outcomes/diseases/drugs** | **Codes** |
| --- | --- | --- |
| Outcomes | Major adverse cardiovascular events (MACE) | ICD-10: I20.0, I21, I22, I50, I60, I61, I63 |
|  | Major adverse kidney events (MAKE) | ICD-10: N18.6, Z99.2  Lab values:8001(eGFR<15)  SNOMED:302497006  CPT:90945, 90937, 90947, 90935, 1012740,1012752 |
|  | Acute coronary syndrome | ICD-10: I20.0, I21, I22 |
|  | Heart failure | ICD-10: I50 |
|  | Stroke | ICD-10: I60, I61, I63 |
|  | Acute kidney injury | ICD-10: N17 |
|  | Depression | ICD-10: F32 |
|  | Suicidal ideation/attempt | ICD-10: R45.851, T14.91 |
|  | Substance use disorders | ICD-10: F10-F19 |
|  | Acute pancreatitis | ICD-10: K85 |
|  | Hypoglycemia | ICD-10: E11.64, E15, E16.0-16.2, E16.A |
|  | Gastrointestinal symptoms | ICD-10: R10-R19 |
| Negative control outcomes | Dog bites | ICD-10: W54.0 |
|  | Nail disorders | ICD-10: L60 |
|  | Ears and hearing test without abnormal findings | ICD-10: Z01.10 |
|  | Sebaceous cyst | ICD-10: L72.3 |
| GLP-1RAs approved for weight loss | Semaglutide | RxNorm: 1991302 |
|  | Liraglutide | RxNorm: 475968 |
|  | Tirzepatide | RxNorm: 2601723 |
| Other anti-obesity medications | Orlistat | RxNorm: 37925 |
|  | Naltrexone | RxNorm: 7243 |
|  | Burpropion | RxNorm: 42347 |
|  | Phentermine | RxNorm: 8152 |
|  | Topiramate | RxNorm: 38404 |
|  | Lorcaserin | RxNorm: 1300701 |
| Inclusion and exclusion criteria | Diabetes | ICD-10: E08-E13 |
|  | Insulins | ATC: A10A |
|  | Biguanides | ATC: A10BA |
|  | SGLT2i | ATC: A10BK |
|  | DPP4i | ATC: A10BH |
|  | Sulfonylureas | ATC: A10BB |
|  | Thiazolidinediones | ATC: A10BG |
|  | GLP-1RA | ATC: A10BJ |
|  | Albiglutide | RxNorm: 1534763 |
|  | Dulaglutide | RxNorm: 1551291 |
|  | Exenatide | RxNorm: 60548 |
|  | Lixisenatide | RxNorm: 1440051 |

ICD-10, International Classification of Diseases, Tenth Revision; ATC, Anatomical Therapeutic Chemical; GLP-1RAs, glucagon-like peptide-1 receptor agonists; AOMs, anti-obesity medications; SGLT2i, sodium glucose cotransporter 2 inhibitors; DPP4i, dipeptidyl peptidase-4 inhibitors; MACE included acute coronary syndrome, heart failure, and stroke; MAKE included end-stage kidney disease and dialysis.

**Table S3. Details of cohort construction**

| **A. GLP-1RA cohort construction** | **Patients** | | **HCOs** |
| --- | --- | --- | --- |
| **Base Population** | 124,298,709 | | 70 |
| **Group 1A: Include: patient using GLP1RA** - The terms in this group occurred on or after Jul 01, 2021. **Must Have:** RxNorm 1991302 semaglutide [≥ 18 years] OR RxNorm 475968 liraglutide [≥ 18 years] OR RxNorm 2601723 tirzepatide [≥ 18 years]  **Group 1B - Any instance of Group 1B occurred within 1 year on or before the first instance of Include: patient using GLP1RA. Must Have:** TNX Curated 9083 BMI [≥ 30 kg/m2, most recent value] OR ICD-10-CM Z68.3 Body mass index [BMI] 30-39, adult OR ICD-10-CM Z68.4 Body mass index [BMI] 40 or greater, adult OR ICD-10-CM E66.01 Morbid (severe) obesity due to excess calories **Cannot Have:** ICD-10-CM E08-E13 Diabetes mellitus ATC A10A INSULINS AND ANALOGUES ATC A10BK Sodium-glucose co-transporter 2 (SGLT2) inhibitors ATC A10BH Dipeptidyl peptidase 4 (DPP-4) inhibitors ATC A10BA Biguanides ATC A10BB Sulfonylureas ATC A10BF Alpha glucosidase inhibitors ATC A10BG Thiazolidinediones ICD-10-CM N18.6 End stage renal disease ICD-10-CM Z99.2 Dependence on renal dialysis RxNorm 37925 orlistat RxNorm 7243 naltrexone RxNorm 42347 bupropion RxNorm 8152 phentermine RxNorm 38404 topiramate RxNorm 1300701 lorcaserin RxNorm 1534763 albiglutide RxNorm 1551291 dulaglutide RxNorm 60548 exenatide RxNorm 1440051 lixisenatide ICD-10-CM I20.0 Unstable angina ICD-10-CM I21 Acute myocardial infarction ICD-10-CM I22 Subsequent ST elevation (STEMI) and non-ST elevation (NSTEMI) myocardial infarction ICD-10-CM I50 Heart failure ICD-10-CM I60 Nontraumatic subarachnoid hemorrhage ICD-10-CM I61 Nontraumatic intracerebral hemorrhage ICD-10-CM I63 Cerebral infarction ICD-10-CM N17 Acute kidney failure ICD-10-CM F32 Depressive episode ICD-10-CM R45.851 Suicidal ideations ICD-10-CM T14.91 Suicide attempt ICD-10-CM F10-F19 Mental and behavioral disorders due to psychoactive substance use ICD-10-CM K85 Acute pancreatitis ICD-10-CM E11.64 Type 2 diabetes mellitus with hypoglycemia ICD-10-CM E15 Nondiabetic hypoglycemic coma ICD-10-CM E16.0 Drug-induced hypoglycemia without coma ICD-10-CM E16.1 Other hypoglycemia ICD-10-CM E16.2 Hypoglycemia, unspecified ICD-10-CM E16.A Hypoglycemia level ICD-10-CM R10-R19 Symptoms and signs involving the digestive system and abdomen CPT 1012740 Dialysis Services and Procedures SNOMED 302497006 Hemodialysis CPT 1012752 Hemodialysis Procedures CPT 90945 Dialysis procedure other than hemodialysis (eg, peritoneal dialysis, hemofiltration, or other continuous renal replacement therapies), with single evaluation by a physician or other qualified health care professional CPT 90937 Hemodialysis procedure requiring repeated evaluation(s) with or without substantial revision of dialysis prescription CPT 90947 Dialysis procedure other than hemodialysis (eg, peritoneal dialysis, hemofiltration, or other continuous renal replacement therapies) requiring repeated evaluations by a physician or other qualified health care professional, with or without substantial revision of dialysis prescription CPT 90935 Hemodialysis procedure with single evaluation by a physician or other qualified health care professional TNX Curated 8001 Glomerular filtration rate/1.73 sq M.predicted [Volume Rate/Area] in Serum, Plasma or Blood by Creatinine-based formula (MDRD) [AND ≤ 15 mL/min/{1.73_m2}, most recent value] TNX Curated 9037 Hemoglobin A1c/Hemoglobin.total in Blood [≥ 6.5 %, most recent value] | | 224,712 | 66 |
| **Group 2A: Include: patient using GLP1RA -** The terms in this group occurred on or after Jul 01, 2021. **Must Have:** RxNorm 1991302 semaglutide [≥ 18 years] OR RxNorm 475968 liraglutide [≥ 18 years] OR RxNorm 2601723 tirzepatide [≥ 18 years]  **Group 2B -** Any instance of Group 2B occurred within 1 day and 1 year before the first instance of Include: patient using GLP1RA. **Must Have:** Visit **Cannot Have:** RxNorm 1991302 semaglutide RxNorm 475968 liraglutide RxNorm 2601723 tirzepatide | 174,957 | | 66 |
| **Final population** | **174,957** | | **66** |

| **B. Other AOM cohort construction** | **Patients** | **HCOs** |
| --- | --- | --- |
| **Base Population** | 124,298,709 | 70 |
| **Group 1A: Include: patient using Other_A... -** The terms in this group occurred on or after Jul 01, 2021. **Must Have:** RxNorm 37925 orlistat [≥ 18 years] OR RxNorm 7243 naltrexone [≥ 18 years] OR RxNorm 42347 bupropion [≥ 18 years] OR RxNorm 8152 phentermine [≥ 18 years] OR RxNorm 38404 topiramate [≥ 18 years] OR RxNorm 1300701 lorcaserin [≥ 18 years]  **Group 1B -** Any instance of Group 1B occurred within 1 year on or before the first instance of Include: patient using Other_A.... **Must Have:** TNX Curated 9083 BMI [≥ 30 kg/m2, most recent value] OR ICD-10-CM Z68.3 Body mass index [BMI] 30-39, adult OR ICD-10-CM Z68.4 Body mass index [BMI] 40 or greater, adult OR ICD-10-CM E66.01 Morbid (severe) obesity due to excess calories **Cannot Have:** ICD-10-CM E08-E13 Diabetes mellitus ATC A10A INSULINS AND ANALOGUES ATC A10BK Sodium-glucose co-transporter 2 (SGLT2) inhibitors ATC A10BH Dipeptidyl peptidase 4 (DPP-4) inhibitors ATC A10BA Biguanides ATC A10BB Sulfonylureas ATC A10BF Alpha glucosidase inhibitors ATC A10BG Thiazolidinediones ICD-10-CM N18.6 End stage renal disease ICD-10-CM Z99.2 Dependence on renal dialysis ATC A10BJ Glucagon-like peptide-1 (GLP-1) analogues RxNorm 2601723 tirzepatide ICD-10-CM I21 Acute myocardial infarction ICD-10-CM I22 Subsequent ST elevation (STEMI) and non-ST elevation (NSTEMI) myocardial infarction ICD-10-CM I20.0 Unstable angina ICD-10-CM I61 Nontraumatic intracerebral hemorrhage ICD-10-CM I63 Cerebral infarction ICD-10-CM I60 Nontraumatic subarachnoid hemorrhage ICD-10-CM I50 Heart failure ICD-10-CM F32 Depressive episode ICD-10-CM R45.851 Suicidal ideations ICD-10-CM T14.91 Suicide attempt ICD-10-CM F10-F19 Mental and behavioral disorders due to psychoactive substance use ICD-10-CM K85 Acute pancreatitis ICD-10-CM E11.64 Type 2 diabetes mellitus with hypoglycemia ICD-10-CM E15 Nondiabetic hypoglycemic coma ICD-10-CM E16.0 Drug-induced hypoglycemia without coma ICD-10-CM E16.1 Other hypoglycemia ICD-10-CM E16.2 Hypoglycemia, unspecified ICD-10-CM E16.A Hypoglycemia level ICD-10-CM R10-R19 Symptoms and signs involving the digestive system and abdomen CPT 1012740 Dialysis Services and Procedures SNOMED 302497006 Hemodialysis CPT 1012752 Hemodialysis Procedures CPT 90945 Dialysis procedure other than hemodialysis (eg, peritoneal dialysis, hemofiltration, or other continuous renal replacement therapies), with single evaluation by a physician or other qualified health care professional CPT 90937 Hemodialysis procedure requiring repeated evaluation(s) with or without substantial revision of dialysis prescription CPT 90947 Dialysis procedure other than hemodialysis (eg, peritoneal dialysis, hemofiltration, or other continuous renal replacement therapies) requiring repeated evaluations by a physician or other qualified health care professional, with or without substantial revision of dialysis prescription CPT 90935 Hemodialysis procedure with single evaluation by a physician or other qualified health care professional TNX Curated 8001 Glomerular filtration rate/1.73 sq M.predicted [Volume Rate/Area] in Serum, Plasma or Blood by Creatinine-based formula (MDRD) [AND ≤ 15 mL/min/{1.73_m2}, most recent value] TNX Curated 9037 Hemoglobin A1c/Hemoglobin.total in Blood [≥ 6.5 %, most recent value] | 279,314 | 67 |
| **Group 2A: Include: patient using Other_A... -** The terms in this group occurred on or after Jul 01, 2021. **Must Have:** RxNorm 37925 orlistat [≥ 18 years] OR RxNorm 7243 naltrexone [≥ 18 years] OR RxNorm 42347 bupropion [≥ 18 years] OR RxNorm 8152 phentermine [≥ 18 years] OR RxNorm 38404 topiramate [≥ 18 years] OR RxNorm 1300701 lorcaserin [≥ 18 years]  **Group 2B -** Any instance of Group 2B occurred within 1 day and 1 year before the first instance of Include: patient using Other_A.... **Must Have:** Visit **Cannot Have:** RxNorm 37925 orlistat RxNorm 7243 naltrexone RxNorm 42347 bupropion RxNorm 8152 phentermine RxNorm 38404 topiramate RxNorm 1300701 lorcaserin | 197,061 | 67 |
| **Final population** | **197,061** | **67** |

**Table S4**. Schoenfeld residual test for proportional hazard assumption.

| **Outcomes** | Schoenfeld residual test (**p-value)** |
| --- | --- |
| **Cardiovascular outcomes** |  |
| MACE | 0.91 |
| Acute coronary syndrome | 0.07 |
| Stroke | 0.003 |
| Heart failure | 0.32 |
| **Mortality** |  |
| All-cause mortality | 0.02 |
| **Kidney outcomes** |  |
| MAKE | 0.33 |
| Acute kidney injury | 0.42 |
| **Mental health outcomes** |  |
| Suicidal ideation/attempt | 0.27 |
| Depression | 0.003 |
| Substance use disorder | 0.34 |
| **Safety outcomes** |  |
| Acute pancreatitis | 0.84 |
| Hypoglycemia | 0.75 |
| Gastrointestinal symptoms | <0.001 |
| **Negative control outcomes** |  |
| Dog bites | 0.3 |
| Nail disorders | 0.88 |
| Hearing test | 0.8 |
| Sebaceous cyst | 0.84 |

**Table S5.** Sensitivity analyses of specific GLP-1RAs vs. AOMs and associations with one-year follow-up

| **Outcomes** | **Tirzepatide vs phentermine + topiramate (n=51,633 in each group)** | **Tirzepatide vs naltrexone + bupropion (n=57,429 in each group)** | **Semaglutide vs phentermine + topiramate (n=88,727 in each group)** | **Semaglutide vs naltrexone + bupropion (n=81,207) in each group** | **Up to 1-yr follow up (n=140,295 in each group)** |
| --- | --- | --- | --- | --- | --- |
| **Cardiovascular outcomes** | | | | | |
| MACE | 0.77(0.69,0.87) | 0.69(0.62,0.77) | 0.83(0.77,0.89) | 0.73(0.68,0.78) | 0.72(0.67,0.78) |
| Acute coronary syndrome | 0.84(0.66,1.08) | 0.78(0.62,0.97) | 0.82(0.70,0.95) | 0.69(0.60,0.81) | 0.83(0.70,0.97) |
| Stroke | 0.51(0.40,0.66) | 0.53(0.42,0.68) | 0.71(0.61,0.81) | 0.65(0.57,0.75) | 0.52(0.44,0.61) |
| Heart failure | 0.86(0.74,0.99) | 0.70(0.61,0.81) | 0.86(0.78,0.95) | 0.75(0.69,0.82) | 0.77(0.70,0.86) |
| **Mortality** | | | | | |
| All-cause mortality | 0.51(0.35,0.73) | 0.31(0.22,0.43) | 0.69(0.56,0.85) | 0.35(0.29,0.42) | 0.31(0.24,0.40) |
| **Kidney outcomes** | | | | | |
| MAKE | 0.39(0.28,0.54) | 0.35(0.25,0.48) | 0.74(0.62,0.88) | 0.61(0.51,0.73) | 0.57(0.47,0.69) |
| Acute kidney injury | 0.83(0.71,0.97) | 0.70(0.60,0.82) | 0.77(0.69,0.85) | 0.65(0.59,0.71) | 0.71(0.63,0.79) |
| **Mental health outcomes** | | | | | |
| Suicidal ideation/attempt | 0.57(0.40,0.82) | 0.38(0.27,0.53) | 0.48(0.39,0.59) | 0.41(0.34,0.49) | 0.39(0.30,0.51) |
| Depression | 0.87(0.82,0.92) | 0.46(0.44,0.49) | 0.89(0.86,0.92) | 0.50(0.49,0.52) | 0.61(0.59,0.64) |
| Substance use disorder | 0.70(0.64,0.76) | 0.46,0.43,0.50) | 0.68(0.64,0.71) | 0.50(0.47,0.52) | 0.57(0.54,0.60) |
| **Safety outcomes** | | | | | |
| Acute pancreatitis | 1.19(0.84,1.67) | 1.13(0.82,1.56) | 1.26(1.01,1.56) | 1.03(0.84,1.28) | 1.10(0.85,1.43) |
| Hypoglycemia | 1.35(1.05,1.72) | 1.30(1.02,1.65) | 1.16(1.00,1.34) | 1.20(1.03,1.41) | 1.06(0.87,1.28) |
| Gastrointestinal symptoms | 1.02(0.98,1.06) | 1.00(0.97,1.04) | 1.02(1.00,1.04) | 1.00(0.97,1.02) | 1.03(1.00,1.06) |
| **Negative control outcomes** | | | | | |
| Dog bites | 1.06(0.68,1.64) | 0.68(0.45,1.03) | 1.15(0.90,1.46) | 0.95(0.75,1.21) | 0.85(0.62,1.16) |
| Nail disorders | 0.94(0.80,1.10) | 0.88(0.75,1.03) | 1.13(1.03,1.24) | 1.00(0.91,1.11) | 0.97(0.86,1.08) |
| Hearing test | 1.09(0.65,1.83) | 0.91(0.55,1.49) | 1.15(0.87,1.51) | 1.04(0.77,1.40) | 0.84(0.58,1.21) |
| Sebaceous cyst | 0.91(0.70,1.17) | 0.88(0.68,1.14) | 1.05(0.91,1.22) | 1.10(0.94,1.28) | 0.93(0.78,1.12) |

GLP-1RAs, glucagon-like peptide-1 receptor agonists; AOMs, anti-obesity medications; MACE, major adverse cardiovascular events; MAKE, major adverse kidney events.

**Table S6-1.** Hazard Ratios (95% CI) from subgroup analyses of GLP-1RAs vs. other AOMs

| **Outcomes** | **Age, ≥ 65 yrs**  **(n= 16,994 in each group)** | **Age, < 65 yrs**  **(n=87,855 in each group)** | **Men**  **(n=29,789 in each group)** | **Women**  **(n=74,385 in each group)** | **History of CVD**  **(n=11,365 in each group)** | **Non- history of CVD (n=128,568 in each group)** |
| --- | --- | --- | --- | --- | --- | --- |
| **Cardiovascular outcomes** | | | | | | |
| MACE | 0.84(0.76,0.92) | 0.79(0.72,0.86) | 0.81(0.73,0.90) | 0.78(0.71,0.85) | 0.77(0.70,0.85) | 0.79(0.74,0.85) |
| Acute coronary syndrome | 0.84(0.69,1.03) | 0.76(0.63,0.91) | 0.83(0.68,1.03) | 0.68(0.56,0.83) | 0.81(0.66,0.99) | 0.75(0.65,0.87) |
| Stroke | 0.63(0.52,0.78) | 0.65(0.54,0.78) | 0.55(0.43,0.71) | 0.69(0.59,0.82) | 0.60(0.46,0.79) | 0.68(0.60,0.77) |
| Heart failure | 0.90(0.81,1.01) | 0.86(0.77,0.97) | 0.86(0.76,0.98) | 0.85(0.76,0.94) | 0.77(0.69,0.87) | 0.87(0.79,0.95) |
| **Mortality** | | | | | | |
| All-cause mortality | 0.53(0.42,0.66) | 0.54(0.42,0.69) | 0.57(0.45,0.73) | 0.43(0.34,0.55) | 0.37(0.26,0.53) | 0.53(0.45,0.63) |
| **Kidney outcomes** | | | | | | |
| MAKE | 1.01(0.77,1.33) | 0.56(0.44,0.72) | 0.67(0.51,0.89) | 0.64(0.53,0.77) | 0.59(0.40,0.88) | 0.66(0.56,0.77) |
| Acute kidney injury | 0.73(0.63,0.84) | 0.83(0.73,0.93) | 0.74(0.64,0.86) | 0.78(0.69,0.88) | 0.68(0.57,0.81) | 0.75(0.69,0.83) |
| **Mental health outcomes** | | | | | | |
| Suicidal ideation/attempt | 0.30(0.14,0.65) | 0.48(0.39,0.61) | 0.32(0.21,0.48) | 0.52(0.40,0.67) | 1.00(0.57,1.74) | 0.37(0.30,0.45) |
| Depression | 0.50(0.46,0.55) | 0.66(0.64,0.69) | 0.47(0.44,0.51) | 0.67(0.65,0.70) | 0.55(0.49,0.60) | 0.64(0.62,0.66) |
| Substance use disorder | 0.67(0.59,0.76) | 0.58(0.55,0.62) | 0.53(0.49,0.58) | 0.62(0.58,0.66) | 0.55(0.48,0.64) | 0.57(0.54,0.59) |
| **Safety outcomes** | | | | | | |
| Acute pancreatitis | 0.98(0.59,1.62) | 1.01(0.80,1.29) | 0.74(0.49,1.11) | 1.20(0.92,1.55) | 0.46(0.22,0.97) | 1.17(0.97,1.41) |
| Hypoglycemia | 1.00(0.69,1.44) | 1.18(0.99,1.40) | 0.93(0.66,1.32) | 1.25(1.04,1.49) | 0.65(0.41,1.02) | 1.12(0.97,1.29) |
| Gastrointestinal symptoms | 0.89(0.84,0.94) | 1.01(0.98,1.03) | 0.98(0.93,1.03) | 0.98(0.96,1.01) | 0.91(0.85,0.97) | 1.00(0.98,1.03) |
| **Negative control outcomes** | | | | | | |
| Dog bites | 0.63(0.30,1.32) | 0.91(0.71,1.17) | 0.84(0.48,1.47) | 0.93(0.71,1.22) | 1.09(0.54,2.22) | 0.82(0.65,1.02) |
| Nail disorders | 0.98(0.81,1.19) | 1.07(0.96,1.19) | 0.97(0.82,1.15) | 1.07(0.95,1.21) | 0.95(0.75,1.21) | 1.06(0.97,1.16) |
| Hearing test | 1.17(0.52,2.64) | 0.88(0.64,1.20) | 0.86(0.44,1.68) | 1.00(0.74,1.37) | 0.82(0.35,1.91) | 0.96(0.72,1.26) |
| Sebaceous cyst | 1.38(0.94,2.00) | 1.15(0.98,1.34) | 1.02(0.78,1.33) | 1.16(0.99,1.36) | 1.22(0.82,1.82) | 1.08(0.94,1.24) |

**Table S6-2.** Hazard Ratios (95% CI) from subgroup analyses of GLP-1RAs vs. other AOMs

| **Outcomes** | **History of CKD (n=2,219 in each group)** | **Non- history of CKD (N=137,866 in each group)** | **BMI, ≥ 30 and < 35 kg/m² (N=55,384 in each group)** | **BMI, ≥ 35.0 and < 40 kg/m² (n=45,763 in each group)** | **BMI, ≥40.0 kg/m² (n=44,321 in each group)** |
| --- | --- | --- | --- | --- | --- |
| **Cardiovascular outcomes** | | | | | |
| MACE | 0.76(0.59,0.97) | 0.76(0.72,0.81) | 0.67(0.61,0.73) | 0.75(0.68,0.83) | 0.75(0.68,0.83) |
| Acute coronary syndrome | 0.64(0.37,1.12) | 0.73(0.65,0.83) | 0.69(0.58,0.83) | 0.74(0.60,0.92) | 0.77(0.62,0.96) |
| Stroke | 0.49(0.26,0.96) | 0.65(0.58,0.74) | 0.66(0.55,0.78) | 0.65(0.54,0.80) | 0.65(0.52,0.82) |
| Heart failure | 0.86(0.65,1.14) | 0.80(0.74,0.86) | 0.63(0.56,0.72) | 0.79(0.69,0.90) | 0.77(0.69,0.87) |
| **Mortality** | | | | | |
| All-cause mortality | 0.34(0.18,0.66) | 0.49(0.42,0.58) | 0.42(0.33,0.53) | 0.44(0.33,0.59) | 0.56(0.43,0.73) |
| **Kidney outcomes** | | | | | |
| MAKE | 0.91(0.62,1.33) | 0.59(0.50,0.69) | 0.65(0.52,0.82) | 0.65(0.51,0.84) | 0.65(0.51,0.83) |
| Acute kidney injury | 0.65(0.51,0.83) | 0.73(0.67,0.80) | 0.74(0.65,0.85) | 0.67(0.58,0.77) | 0.73(0.64,0.84) |
| **Mental health outcomes** | | | | | |
| Suicidal ideation/attempt | 0.27(0.03,2.23) | 0.46(0.38,0.55) | 0.43(0.31,0.59) | 0.40(0.29,0.56) | 0.50(0.37,0.66) |
| Depression | 0.49(0.39,0.62) | 0.64(0.62,0.66) | 0.57(0.54,0.60) | 0.65(0.61,0.68) | 0.71(0.68,0.75) |
| Substance use disorder | 0.64(0.47,0.89) | 0.57(0.54,0.59) | 0.52(0.49,0.56) | 0.57(0.53,0.62) | 0.69(0.64,0.74) |
| **Safety outcomes** | | | | | |
| Acute pancreatitis | 1.42(0.35,5.75) | 1.17(0.97,1.40) | 1.19(0.89,1.60) | 1.13(0.83,1.55) | 1.04(0.76,1.41) |
| Hypoglycemia | 0.71(0.19,2.71) | 1.17(1.02,1.34) | 1.23(0.98,1.56) | 1.07(0.84,1.36) | 1.08(0.87,1.34) |
| Gastrointestinal symptoms | 0.91(0.79,1.05) | 1.00(0.98,1.02) | 0.99(0.96,1.02) | 1.00(0.97,1.03) | 0.99(0.96,1.02) |
| **Negative control outcomes** | | | | | |
| Dog bites | 0.44(0.09,2.19) | 0.85(0.69,1.06) | 0.87(0.62,1.24) | 1.05(0.75,1.47) | 1.07(0.75,1.55) |
| Nail disorders | 0.59(0.32,1.07) | 1.00(0.92,1.09) | 0.94(0.83,1.07) | 0.93(0.81,1.07) | 1.06(0.92,1.22) |
| Hearing test | 0.35(0.04,3.08) | 0.92(0.71,1.20) | 0.75(0.51,1.12) | 1.10(0.71,1.70) | 0.93(0.58,1.49) |
| Sebaceous cyst | 1.39(0.50,3.87) | 1.08(0.95,1.23) | 1.01(0.82,1.25) | 1.04(0.84,1.29) | 1.23(0.99,1.53) |

GLP-1RAs, glucagon-like peptide-1 receptor agonists; AOMs, anti-obesity medications; CVD, cardiovascular disease; CKD, chronic kidney disease; BMI, body mass index; MACE, major adverse cardiovascular events; MAKE, major adverse kidney events.

**Figure S1.** Love plots of propensity score distributions before and after matching in sensitivity and subgroup analyses.

**Tirzepatide vs phentermine + topiramate**


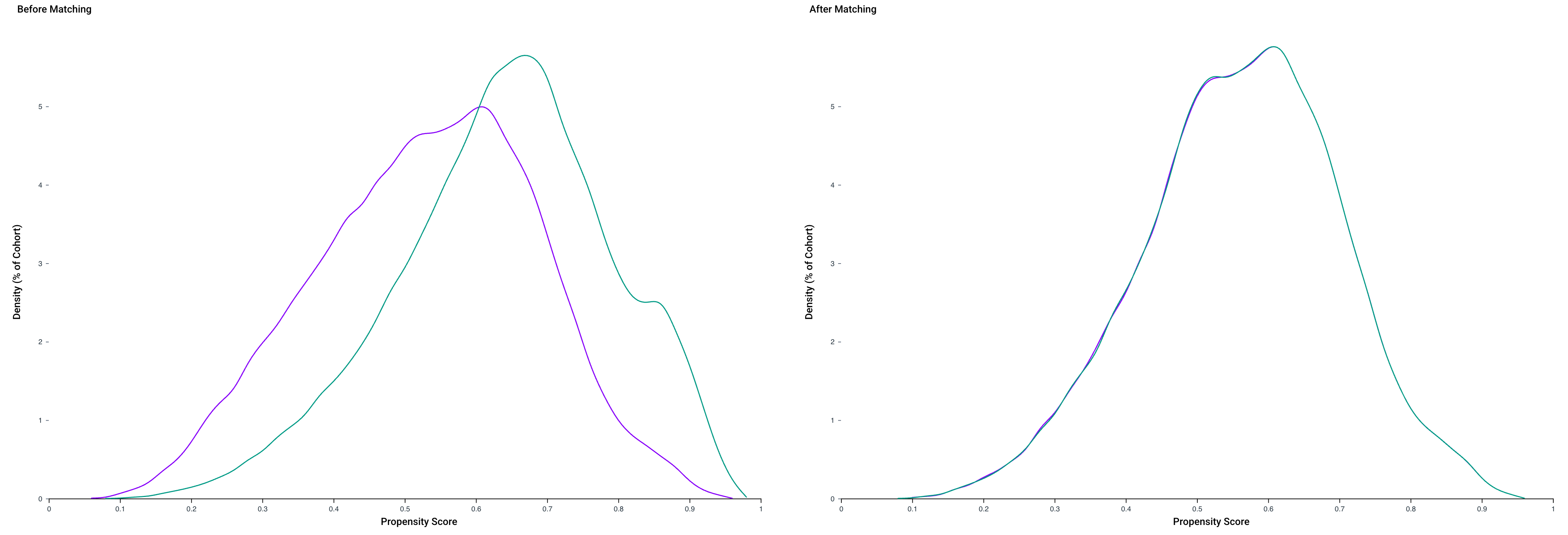


**Tirzepatide vs naltrexone + bupropion**


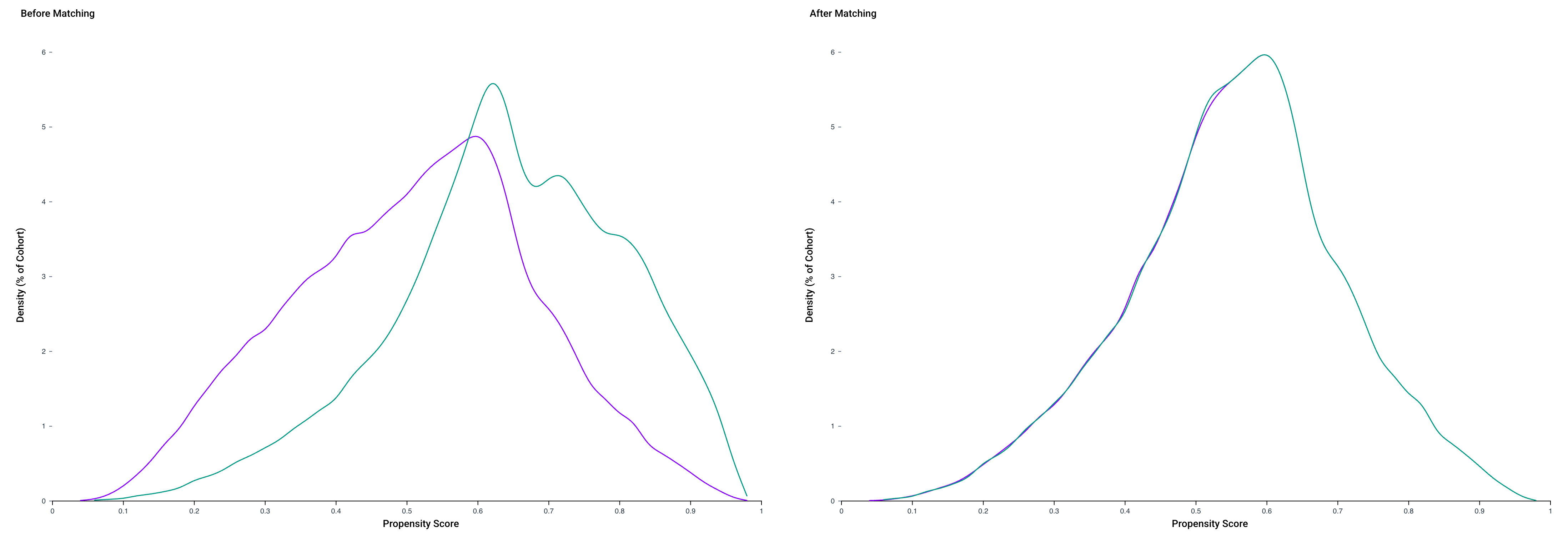


**Semaglutide vs phentermine + topiramate**


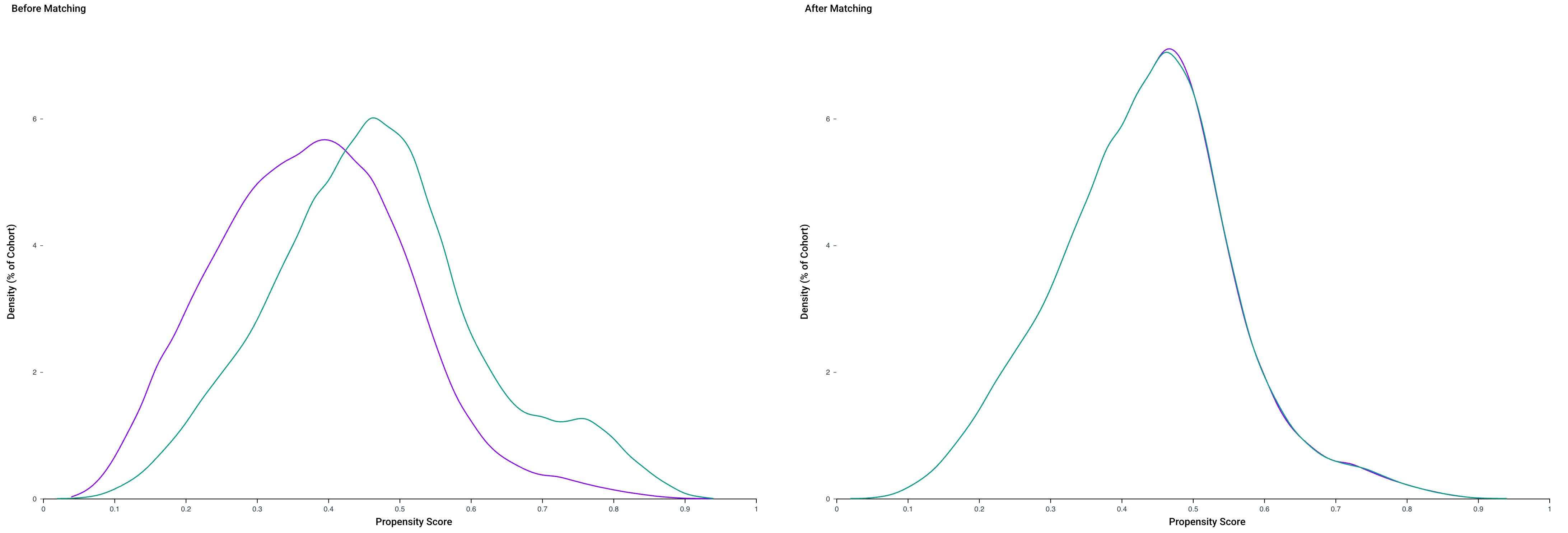


**Semaglutide vs naltrexone + bupropion**

**
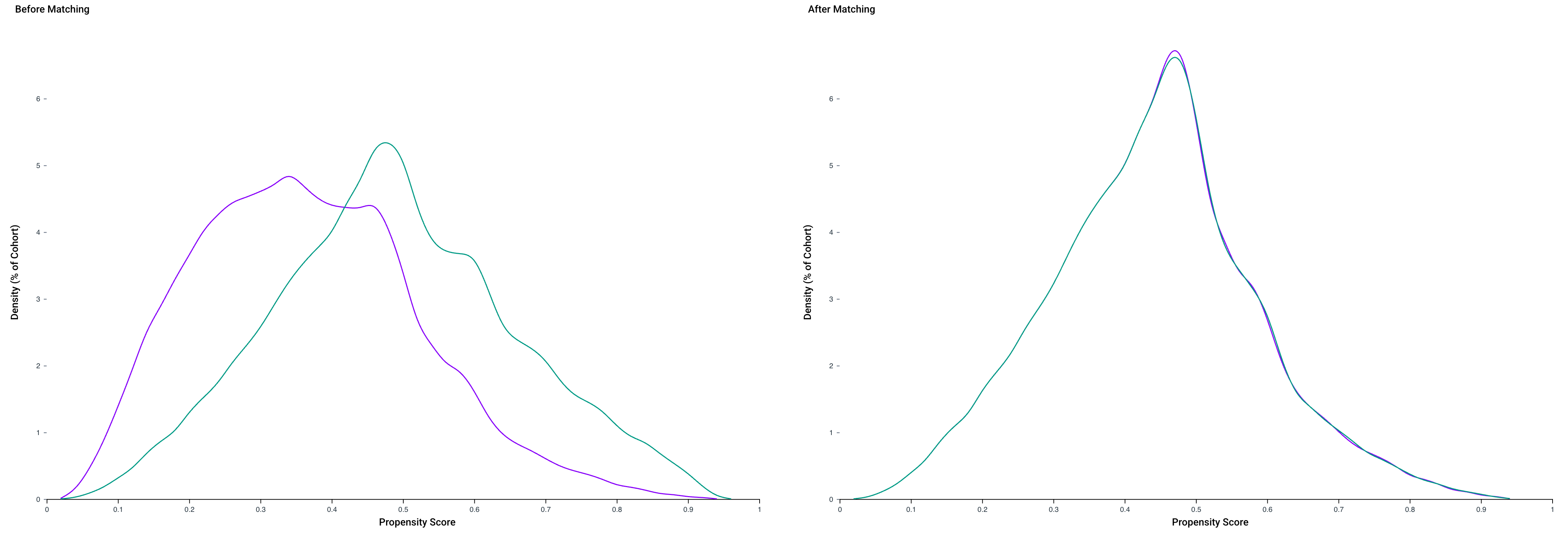
**

**Up to 1-year follow-up**

**
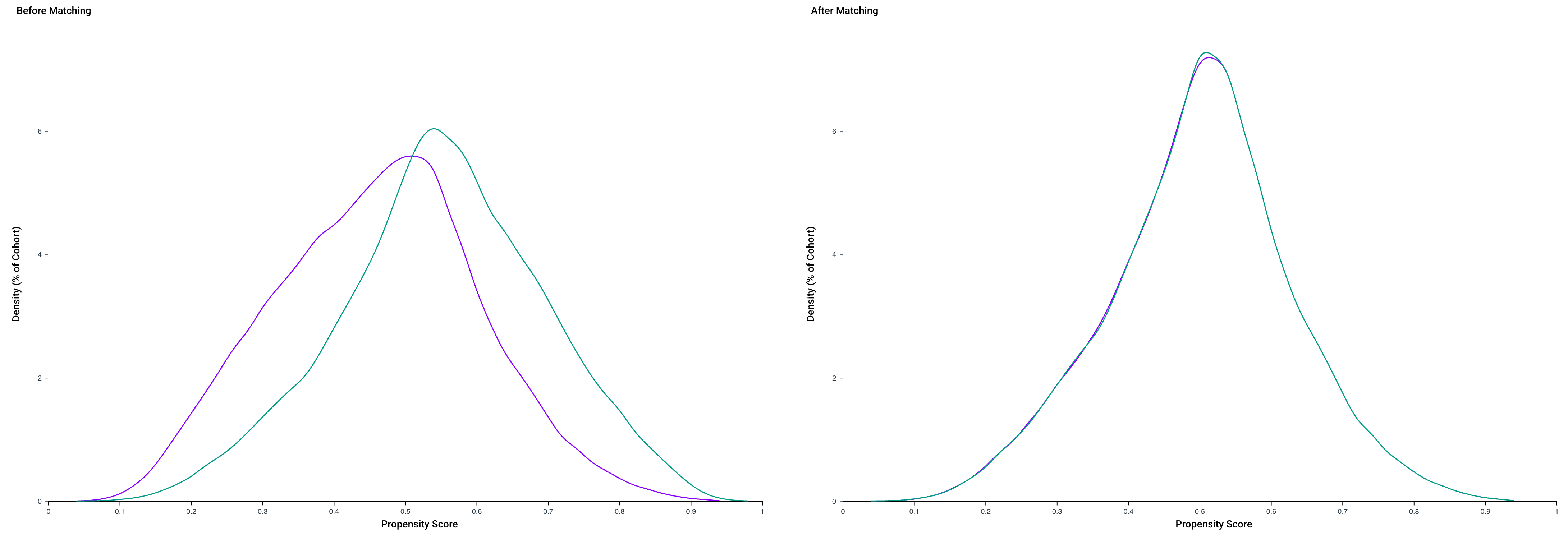
**

**Age, ≥ 65 yrs**

**
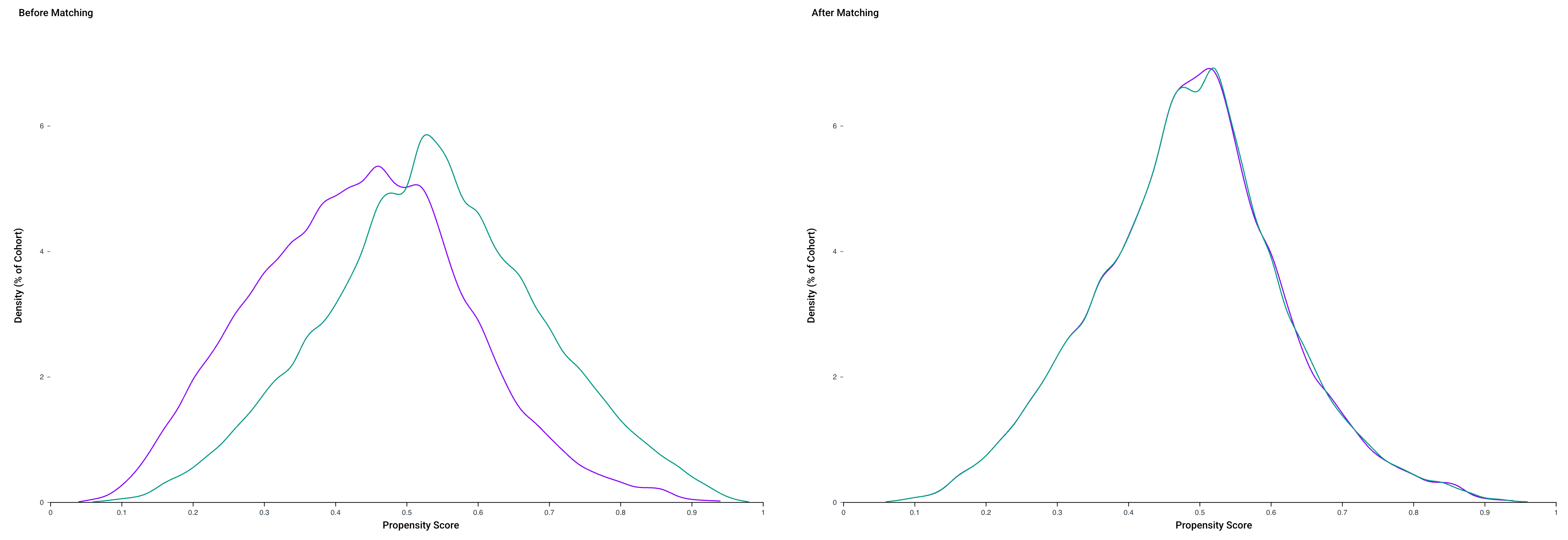
**

**Age, < 65 yrs**

**
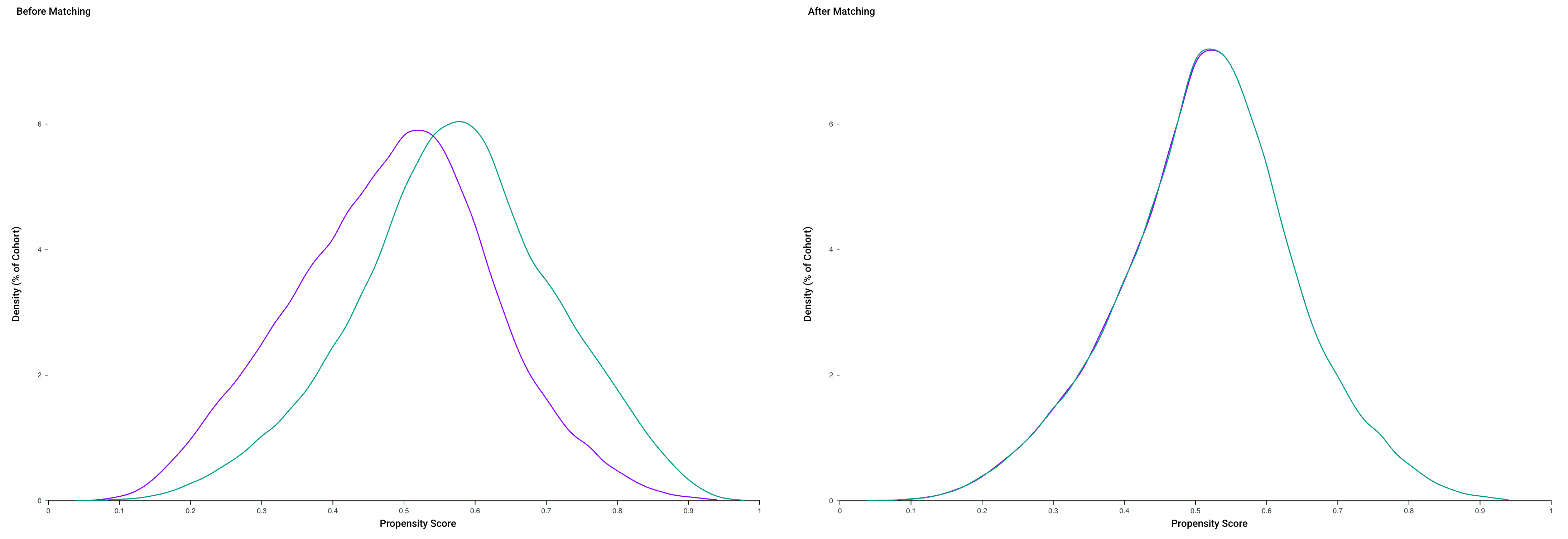
**

**Men**

**
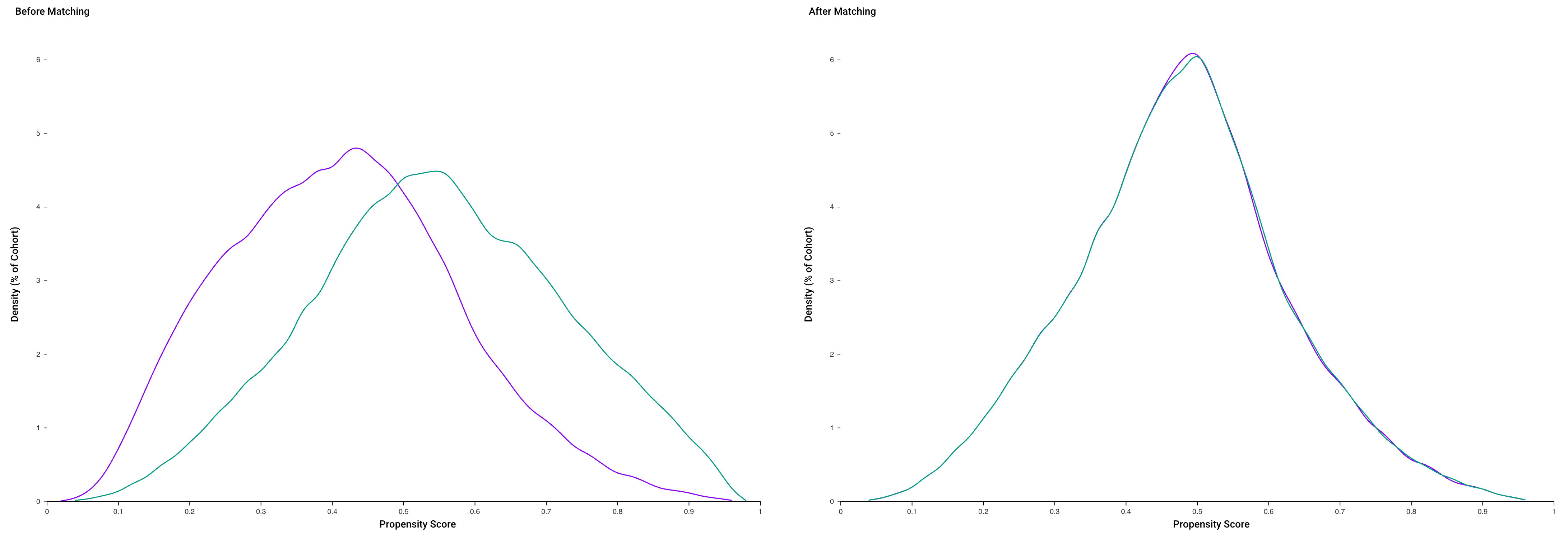
**

**Women**

**
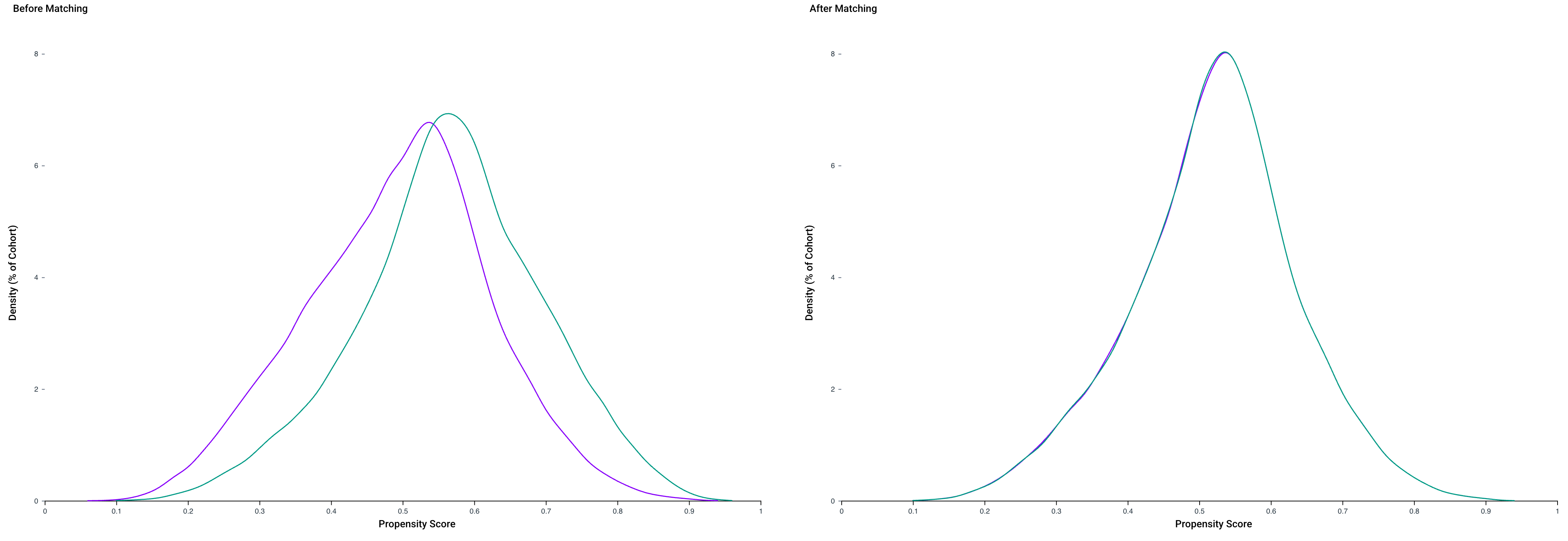
**

**History of CVD**

**
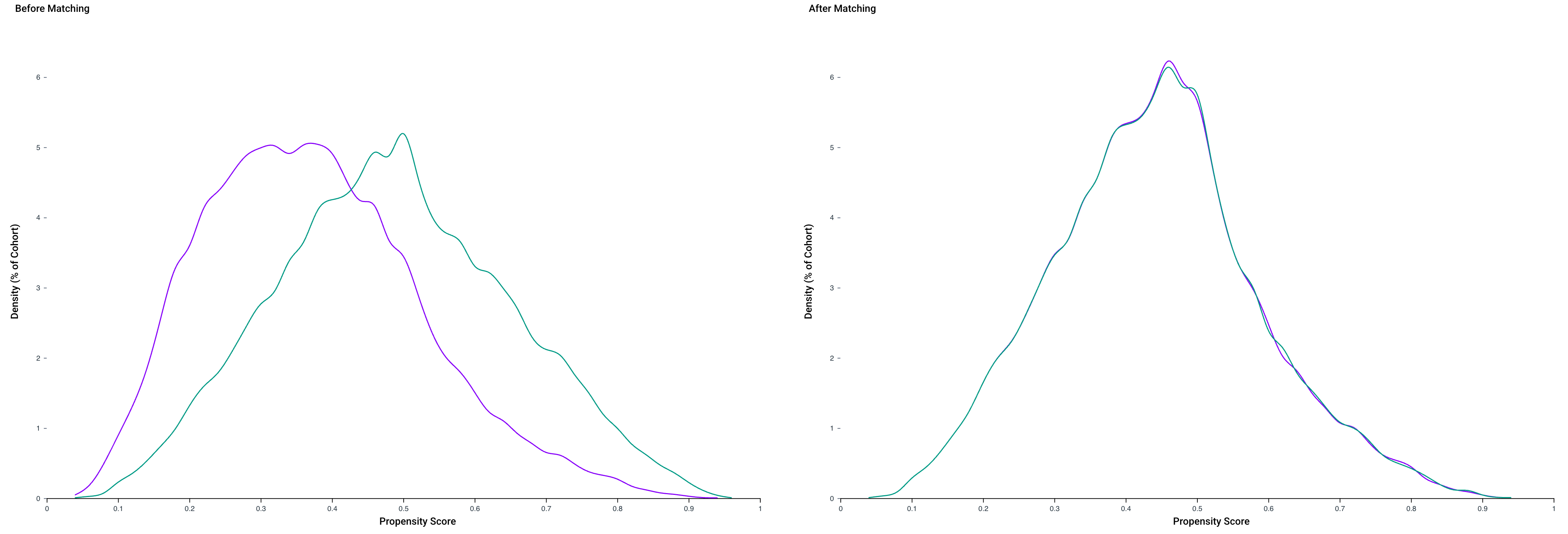
**

**Non-history of CVD**

**
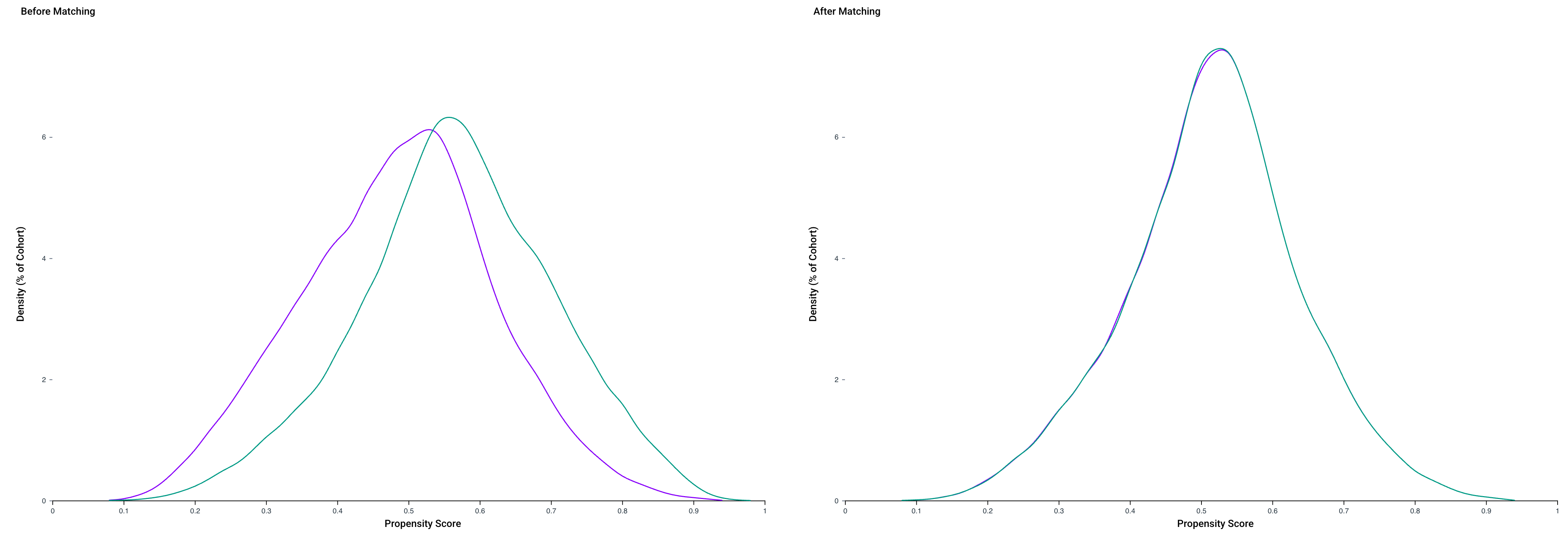
**

**History of CKD**

**
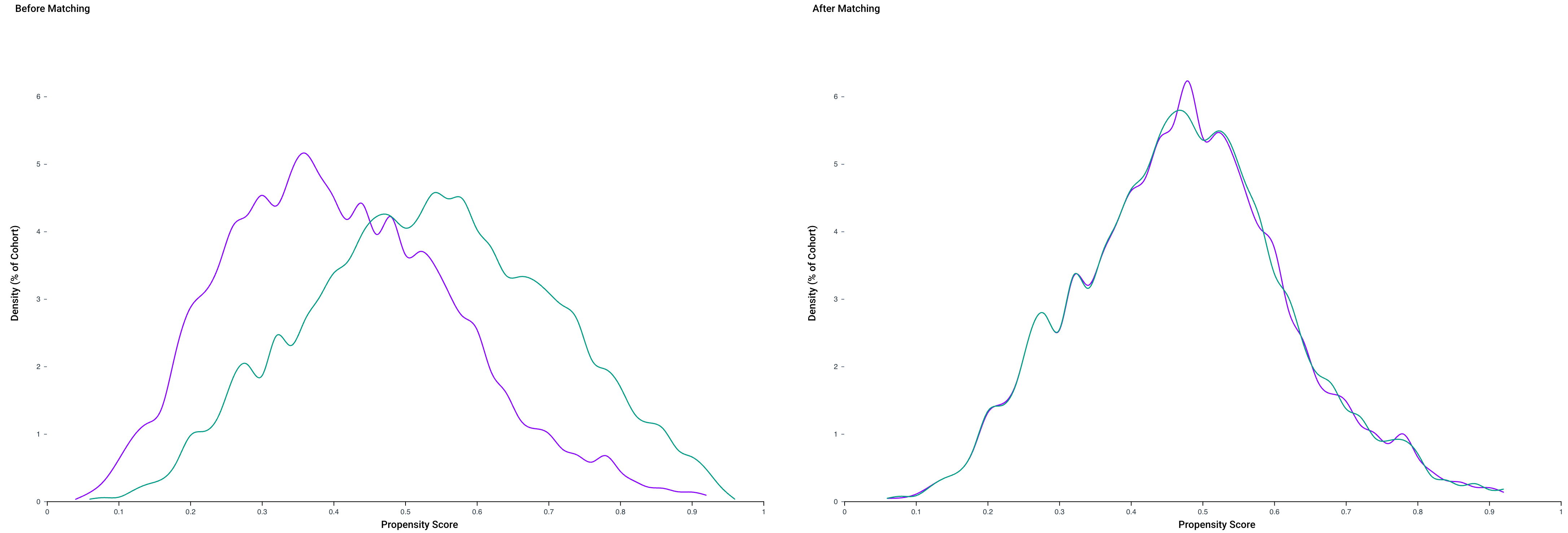
**

**Non-history of CKD**

**
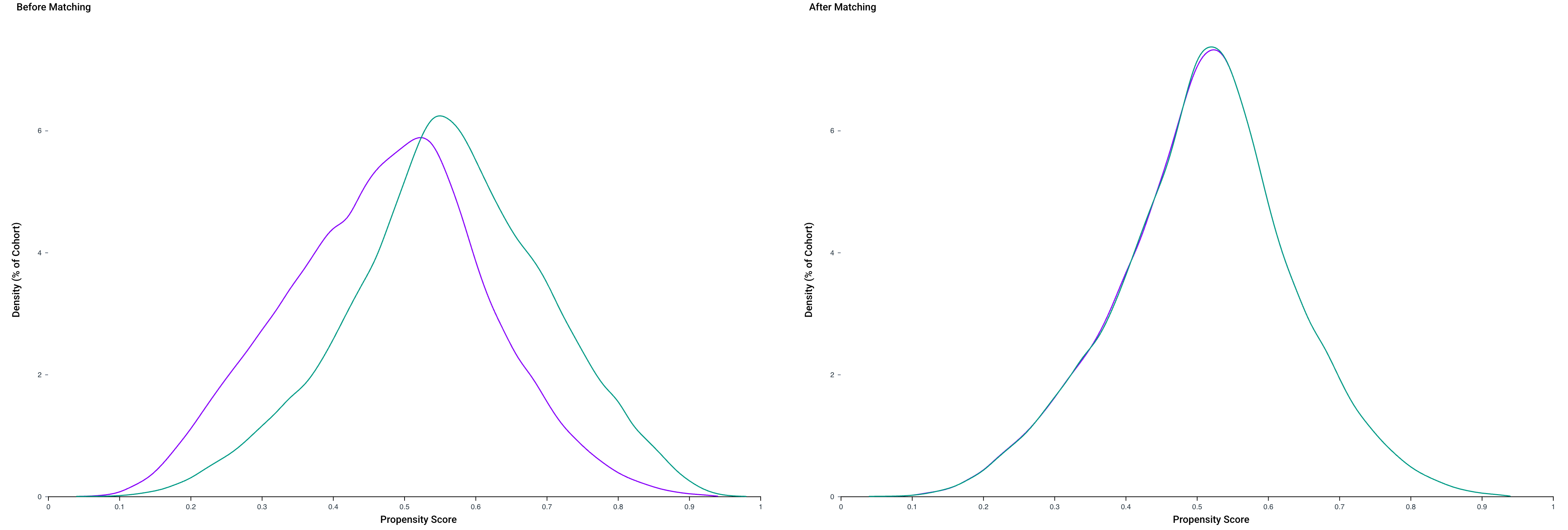
**

**BMI, ≥ 30 and < 35 kg/m²**

**
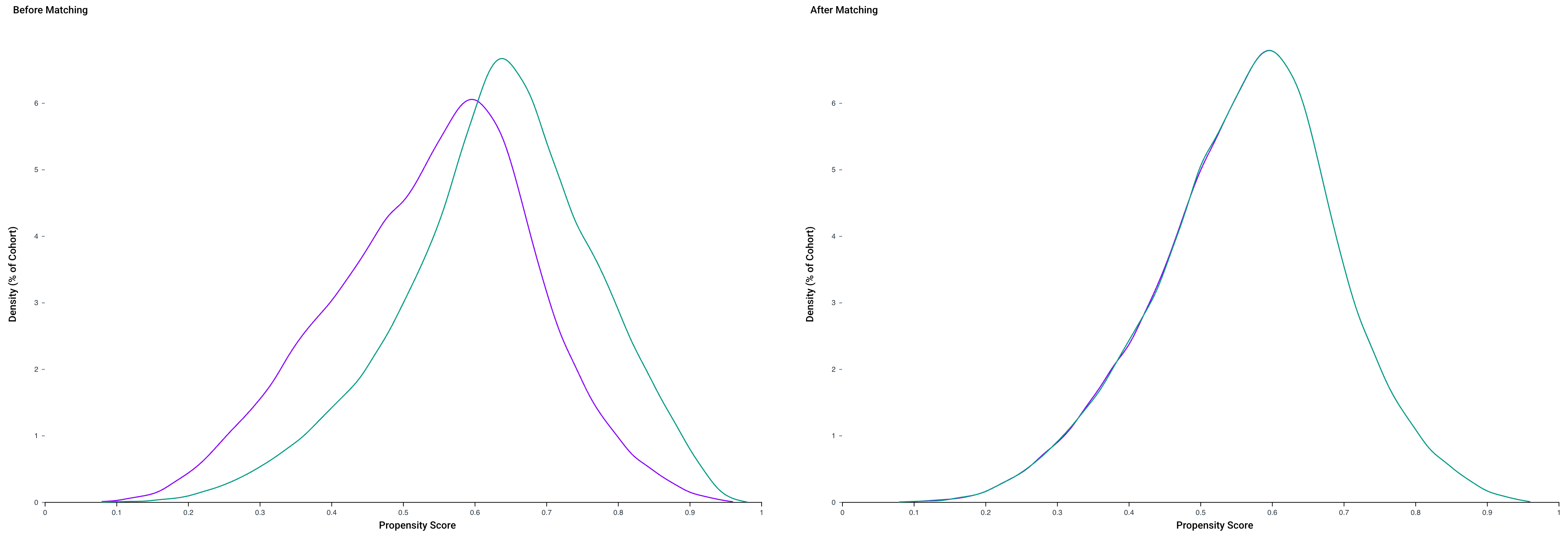
**

**BMI, ≥ 35.0 and < 40 kg/m²**

**
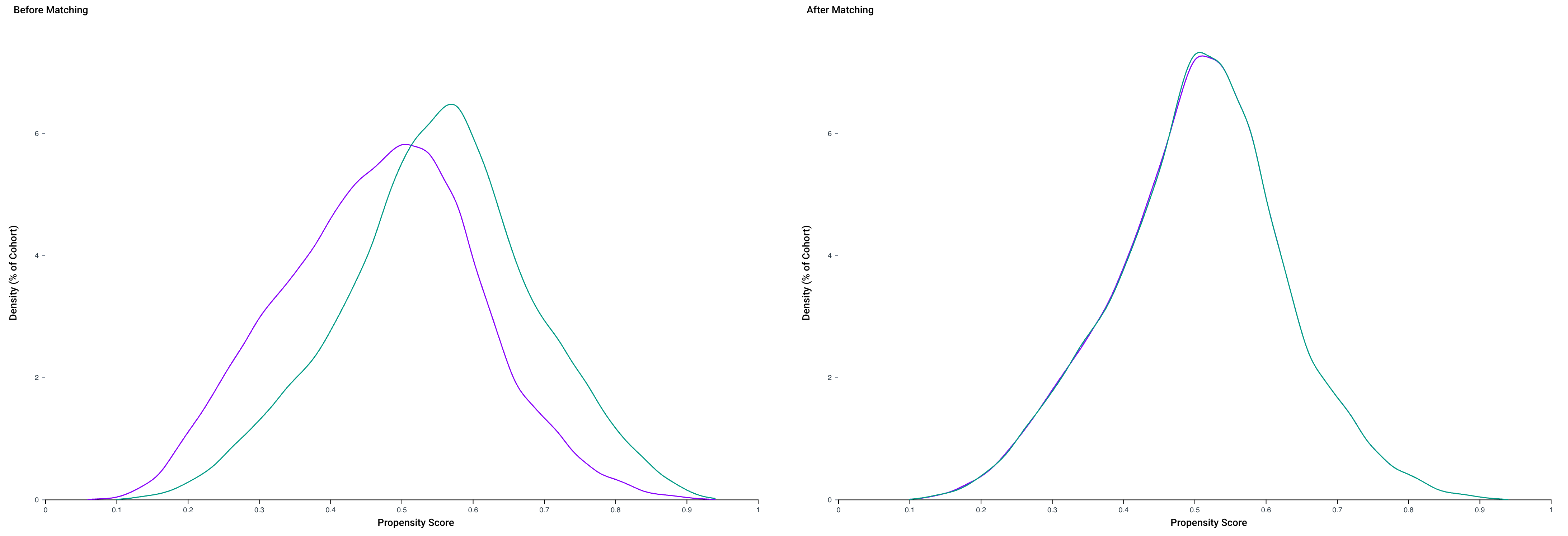
**

**BMI, ≥40.0 kg/m²**


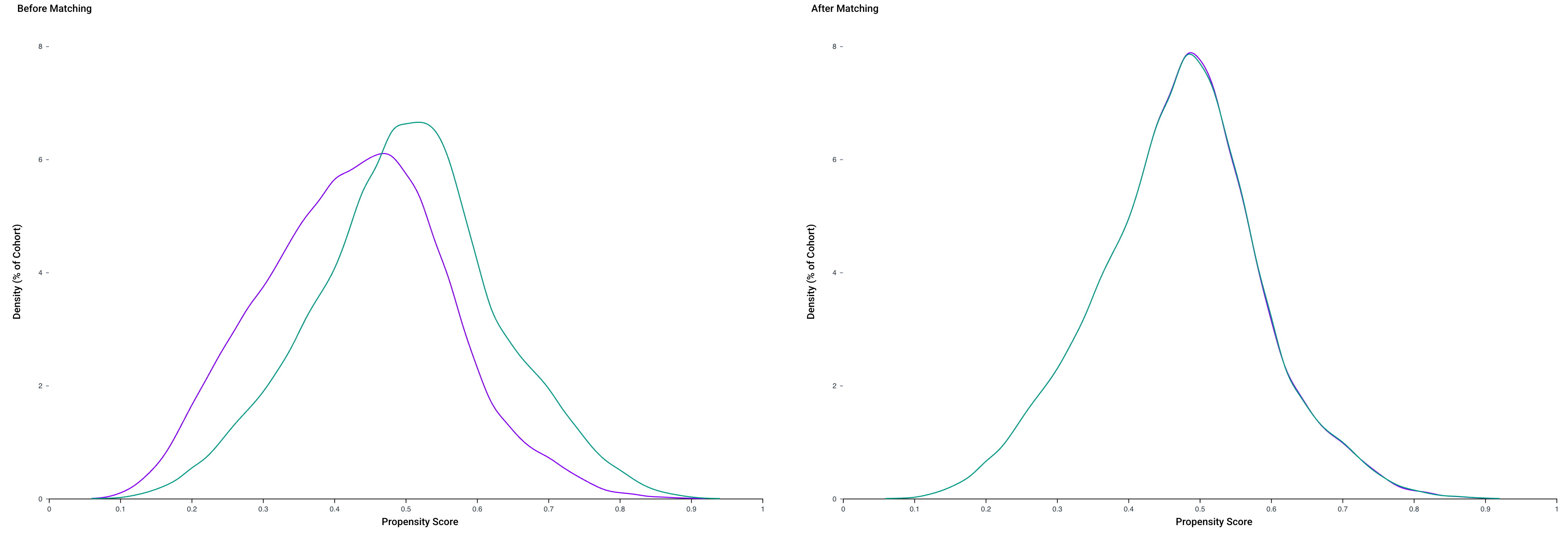

Supplement: Supplementary file 1 — Table S1. Target trials emulation. Table S2. Codes used to identify outcomes, diseases, and drugs. Table S3. Details of cohort construction. Table S4. Schoenfeld residual test for proportional hazard assumption. Table S5. Sensitivity analyses of specific GLP‐1RAs versus AOMs and associations with 1‐year follow‐up. Table S6. Hazard Ratios (95% CI) from subgroup analyses of GLP‐1RAs versus other AOMs. Figure S1. Love plots of propensity score distributions before and after matching in sensitivity and subgroup analyses. [file DOM-27-6527-s001.docx]
